# Quantum-entangled feature selection and spiking graph transformer networks for early detection of childhood behavioral markers

**DOI:** 10.3389/fnbeh.2026.1797210

**Published:** 2026-05-22

**Authors:** Karthiga M, Emerson Raja Joseph, Subhash Patil, Saranya K

**Affiliations:** 1 Department of Computer Science and Engineering, Bannari Amman Institute of Technology, Sathyamangalam, Tamil Nadu, India; 2 Postdoctoral Researcher, Multimedia University, Melaka, Malaysia; 3 Faculty of Engineering and Technology Centre for Advanced Analytics, COE of Artificial Intelligence, Multimedia University, Melaka, Malaysia; 4 Department of Surgery, International Medical University, Management and Science University, Shah Alam, Malaysia; 5 Department of Computer Science and Engineering, Bannari Amman Institute of Technology, Sathyamangalam, Tamil Nadu, India

**Keywords:** childhood behavioral markers, graph transformer, neuromorphic computing, quantum variational feature selection, sleep-wake detection, spiking neural networks, wearable accelerometers

## Abstract

**Introduction:**

Early identification of childhood behavioural markers using wearable sensing is important for timely intervention in developmental and sleep-related disorders. Wrist-worn accelerometer data provide objective measures of behavioural regulation by capturing actigraphy-derived states such as Sleep, Wake, and Transitional periods. However, existing deep learning methods for behavioural state detection often face challenges related to redundant features, sensitivity to sensor noise, limited robustness in long-term wearable deployment, and high energy consumption.

**Methods:**

This study proposes a Quantum Variational Feature Selection-Spiking Graph Transformer Network (QVFS-SGTN), a hybrid quantum-neuromorphic framework for robust and energy-efficient behavioural state classification. The prediction task was defined as a three-class classification problem involving Sleep, Wake, and Transitional behavioural states from high-frequency wrist-worn accelerometer data. The proposed model integrates a parameterized quantum circuit-based feature selector to identify non-linear and entangled sensor correlations. Selected features are then processed by a spiking graph transformer network, which models temporal dependencies through event-driven self-attention and neuromorphic neuron dynamics. Experiments were conducted using the Child Mind Institute wearable dataset, with additional cross-dataset validation performed on the external Multi-Ethnic Study of Atherosclerosis sleep dataset.

**Results:**

The proposed QVFS-SGTN framework achieved state-of-the-art performance on the Child Mind Institute wearable dataset, with an accuracy of 0.968, an F1-score of 0.968, and an AUC of 0.991. Robustness evaluation demonstrated stable performance under significant Gaussian noise, maintaining accuracy above 92%. Energy analysis showed a 40–55% reduction in computational cost compared with conventional deep learning models. In cross-dataset evaluation using the MESA sleep dataset, the model achieved an accuracy of 0.931 without fine-tuning, indicating strong generalization capability.

**Discussion:**

The findings demonstrate that combining quantum-enhanced feature selection with spiking graph-based temporal modelling can improve the robustness, accuracy, and energy efficiency of wearable behavioural state detection. The QVFS-SGTN framework effectively addresses key limitations of existing deep learning approaches, including feature redundancy, sensor noise sensitivity, and computational cost. These results support the potential of the proposed hybrid quantum-neuromorphic model for scalable, long-term, real-world paediatric behavioural monitoring.

## 1 Introduction

The introduction of wearable sensing technologies has radically transformed the way behavior and sleep are monitored through the ability of non-invasive continuous data recording in natural, real-world contexts (Guillot and Thorey, 2021; Supratak and Guo, 2020). Multivariate motion responses that encode fine postural responses and activity patterns associated with sleep-wake transitions and behavioral states are generated by accelerometers at the wrist, in particular (Biswal et al., 2018; Chambon et al., 2018). The early detection of these behavioral manifestations in childhood is of vital clinical significance: early childhood is a developmental period when the absence of irregularities in sleep patterns, activity rhythms, and behavioral transitions are closely related to the development of neurodevelopmental conditions like Autism Spectrum Disorder (ASD), Attention Deficit Hyperactivity Disorder (ADHD), and insomnia in children (Chen et al., 2015; Vial and Almon, 2023). Early detection of these markers can allow clinicians to take early therapeutic action, modify behavioral management practices, track treatment efficacy over time and enhance long-term cognitive and behavioral outcomes (Chambon et al., 2018; Guillot and Thorey, 2021). Also, wearable monitoring, which is performed continuously, is a scalable, low-burden alternative to in-clinic polysomnography, which is particularly useful in the large-scale screening of pediatrics in resource-constrained environments (Guillot et al., 2020; Supratak and Guo, 2020). However, it is difficult to extract credible behavioral measurements based on raw wearable measurements because of sensor noise, inter-axis redundancy, and the non-linearity of human movement (Jia et al., 2021; Wu et al., 2020).

One of the most dependable and measurable predictors of childhood behavioral wellness are sleep-wake dynamics (Biswal et al., 2018; Chambon et al., 2018). The differences in sleep onset latency, awakenings during the night, stability of the circadian rhythms in sleep, and intensity of movements during sleep are strongly related to the neurodevelopmental and psychological disorders, including attention deficit hyperactivity disorder, autism spectrum disorder, anxiety, and emotional regulation disorders (Chen et al., 2015; Vial and Almon, 2023). Therefore, the sleep stage determination offers objective physiological and behavioral data to supplement the conventional clinical evaluations (Biswal et al., 2018; Guillot et al., 2020). Acceleration-based measurements of postural variations and body movement intensity are proxies of sleep-wake transitions in wearable sensing conditions (Jia et al., 2021; Supratak and Guo, 2020), and allow continuous and non-invasive observation of behavioral patterns in natural conditions (Child Mind and Institute, 2023; Guillot and Thorey, 2021). The proposed framework capitalizes on sleep stage identification as a basic element of early detection of clinically relevant childhood behavioral markers by connecting sleep dynamics and behavioral outcomes. To understand the task of prediction that will be used in this paper, the classification of three behavioral states that are based on actigraphy signals, namely Sleep, Wake, and Transitional, is defined as the prediction task that this paper aims to solve using the signals of the wrist-worn accelerometer. These states are objective and clinically significant circadian and behavioral regulation indicators in children. The term childhood behavioral markers is hence applied all over the manuscript to refer to insights based on such sleep-wake interactions and not as distinct prediction targets.

Childhood behavioral markers as used in this study can be described as objective, measurable indicators of behavioral and circadian regulation that are based on sleep-wake interactions, such as sleep duration, wakefulness dynamics and transitional micro-movements. Such indicators offer clinically informative data on neurodevelopmental and emotional health and cannot be viewed as being reflective of the entire range of behavioral responses to external stimuli. These behavioral states derived as results of actigraphy are widely confirmed in the research of pediatric sleep as the predictors of neurodevelopment and psychological health. It has been observed that sleep continuity abnormalities, nocturnal awakenings, and abnormal circadian rhythms are closely linked with behavioral and emotional abnormalities in children (Biswal et al., 2018; Chambon et al., 2018). Hence, Sleep, Wake, and Transitional states classification offers clinically significant information that goes beyond mere recognizing of activities giving the chance of an objective and scalable evaluation of childhood behavioral health (Chen et al., 2015; Vial and Almon, 2023).

Deep learning has become the new paradigm of automated sleep and behavioral state detection. Initial research showed that deep neural networks are capable of scoring physiological signals at a level comparable to that of experts when it comes to scoring sleep (Biswal et al., 2018). Following architectures added multivariate and multimodal time-series modeling to more effectively model time dependencies (Chambon et al., 2018), and transfer learning methods added robustness and scalability to large datasets (Guillot and Thorey, 2021). Models that use attention also added sensitivity to transient events through the selective emphasis of informative temporal segments (Havlícek et al., 2019; Qu et al., 2020). Lightweight frameworks like TinySleepNet dealt with the computational limitations, which allowed it to be deployed on edge devices (Supratak and Guo, 2020). Benchmarking of automated sleep staging techniques has also been made easier using public datasets, such as the Dreem datasets (Guillot et al., 2020).

Although deep learning has achieved impressive advances in behavioral and sleep state detection, there are a number of inherent weaknesses that limit its usefulness in early detection of behavioral indicators of childhood behavior. Traditional architectures including convolutional and recurrent neural networks are mostly based on dense, continuous-valued representations, and usually require handcrafted or statistically selected features. These methods find it difficult to represent the non-linear and inter-axis interactions found in high-frequency wearable sensor data, resulting in feature overlap and poor discriminative quality. In addition, deep learning architectures tend to be very sensitive to sensor noise and motion artifact that is usually present in practice in pediatric monitoring systems, leading to reduced reliability. The other important weakness is that they have high computational and energy costs, making them difficult to deploy in the long-term on wearable or edge devices. Moreover, most current models have low interpretability and cross-dataset generalization, which makes them less applicable in clinical settings with different populations. These problems underscore the need to have new computational approaches that are able to offer robust feature disentanglement, energy-efficient temporal modeling and strong generalization. The proposed Quantum Variational Feature Selection and Spiking Graph Transformer Network (QVFS-SGTN) framework is expected to overcome these limitations and be able to combine quantum-enhanced feature representation with neuromorphic, event-driven processing in a single architecture.

Graph-based learning has been more recently popularized to learn relational and temporal dependencies on behavioral data. The classification of sleep stages has been successfully implemented in spatial-temporal graph convolutional networks and multi-view graph formulations that are more generalized and robust (Jia et al., 2021). Geometric deep learning and graph neural networks Surveys Geometric deep learning and graph neural networks demonstrate the usefulness of graph representations in modeling structured dependencies between signals and time (Cao et al., 2020; Li et al., 2018; Wu et al., 2020; Xu et al., 2018; Zhang et al., 2019; Zhou et al., 2020). However, the majority of graph-based sleep models are based on dense computations and classical feature extraction pipelines, which makes them less suitable to use in long-term wearable applications.

Simultaneously, quantum machine learning (QML) has developed as an effective architecture to model non-linear feature interactions with the use of quantum Hilbert space representations. Variational quantum algorithms and quantum neural networks have a higher representational capacity and can learn complex, non-linear relationships in data by embedding classical features into high-dimensional quantum Hilbert spaces and exploiting entanglement to learn correlations that are hard to learn using classical neural networks (Abbas et al., 2021; Cerezo et al., 2021; Mitarai et al., 2018). The re-upload of data and quantum-enhanced feature space allow efficient supervised learning with a small amount of training data (Pérez-Salinas et al., 2020; Schuld and Killoran, 2019). Quantum kernel research and data efficiency studies also indicate the opportunities of quantum models during the noisy intermediate-scale quantum (NISQ) era (Huang et al., 2021; Wang et al., 2021). Complex data distributions have also been studied with quantum generative models (Lloyd and Weedbrook, 2018). Although such advances have been made, QML has mostly been restricted to synthetic benchmarks or small-scale classification problems, and has not been extensively applied to real world wearable sensor data.

The other amount of research that is complementary is the area of neuromorphic computing and spiking neural networks (SNNs). The third generation of neural networks is the spiking models, which are models that use discrete spike events instead of continuous activations (Maass, 1997). Recent progress in surrogate-gradient learning has made it possible to train deep spiking networks (Neftci et al., 2019; Vial and Almon, 2023), and neuromorphic hardware systems like Loihi have shown substantial energy efficiency improvements on temporal processing applications (Davies et al., 2021; Roy et al., 2019). More recent uses of spiking networks have been in action recognition, event-stream classification, and cognitive signal processing (Comsa et al., 2020; Yao et al., 2021; Zhang et al., 2022). There are also graph-based spiking neural networks that are proposed to be used in learning complex relationships (Cai et al., 2024). New photonic neuromorphic systems also hold additional potential with ultra-low-latency and energy-efficient computation (Li et al., 2025).

Despite such developments, the existing solutions are characterized by a number of limitations. Traditional deep learning algorithms are usually computationally-heavy processes, including large-scale convolutional and recurrent layers, leading to high memory and energy usage, making them less usable in the long term wearable application. Moreover, these models are usually based on raw or statistically obtained features, which can have redundant or poorly informative elements, resulting in inefficient learning and possible overfitting. Wearable sensor data at high frequencies are also subject to noise and motion artifacts, and traditional deep learning architectures often do not have mechanisms built in to guarantee resilience to these perturbations. Also, most of the current methods offer limited interpretability and exhibit less generalization to external datasets with different demographic or sensor properties. Together, these issues emphasize the necessity of stronger and more efficient computational models of reliable childhood behavioral marker detection. Graph-based models usually do not have principled refinement mechanisms of features whereas spiking networks usually use low-dimensional, pre-processed inputs. End-to-end temporal modeling pipelines, in turn, are seldom combined with quantum learning methods. Besides, cross-dataset generalization is a significant issue in sleep and behavioral study, as evidenced by population-level differences in datasets like the Multi-Ethnic Study of Atherosclerosis (MESA) (Caro et al., 2022; Chen et al., 2015).

Despite the fact that it is easy to draw a line between sleep and wake states in idealized conditions, the data of actigraphy in the real world has a number of challenges, such as low-amplitude movement in quiet wakefulness, motion artifact, inter-subject variability, and similarity in signal properties between the behavioral states. Transitional movements are especially delicate and time-scarce and thus not easily identified. Therefore, conventional threshold-based or shallow machine learning models can tend to have restricted sensitivity and generalization. The rationale behind the proposed quantum-neuromorphic framework is thus the necessity to learn complex non-linear interactions, learn finer-grained temporal dynamics, and be resilient to diverse heterogeneous sensing environments.

The foregoing review of existing approaches reveals several critical and unresolved limitations that directly motivate the present work. Although great progress has been made in the field of deep learning, graph neural networks, quantum machine learning, and neuromorphic computing, there are many important limitations that have not been addressed yet when it comes to childhood behavioral marker detection. Current deep learning methods tend to be computationally intensive and noise-sensitive to noisy and redundant high-frequency wearable sensor data. Graph-based models are useful in modeling spatial-temporal relationships, but generally do not have principled mechanisms to decompose the complex non-linear relationships among features. Quantum machine learning has strong representational potential; but it has not been well applied to real-world wearable behavioral analysis, or end-to-end temporal modeling. Likewise, neuromorphic and spiking neural networks offer energy-saving event-based processing but typically also need pre-refined low-dimensional inputs and are rarely combined with high-end feature selection methods. Moreover, most of the available systems exhibit less robustness and lower generalization across different datasets with different demographic and sensor profiles. These gaps point to the necessity of a common computational platform, which combines non-linear feature disentanglement, energy-efficient temporal modeling, and powerful cross-dataset generalization. The QVFS-SGTN framework proposed aims to overcome these issues in a unified end-to-end system.

It is based on these shortcomings that this paper introduces a single Quantum Variational Feature Selection and Spiking Graph Transformer Network (QVFS-SGTN) framework to the early identification of childhood behavioral indicators using high-frequency wearable accelerometer data. The main contribution of this work is the combination of quantum-enhanced feature disentanglement and neuromorphic, event-driven graph transformer modeling in one end-to-end architecture. The integrated architecture allows strong recognition of non-linear behavioral patterns and at the same time, strong cross-dataset generalization and energy-efficient temporal processing. The suggested framework defines a new paradigm of computation, scalable and reliable pediatric behavioral monitoring in wearable settings. Besides this main contribution, the study contributes:

(i) a quantum variational mechanism for identifying non-linear and entangled dependencies in wearable sensor features, and

(ii) a spiking graph transformer architecture that facilitates energy-efficient and interpretable temporal modeling of behavioral dynamics.

## 2 Related works

### 2.1 Quantum machine learning for feature representation

Quantum machine learning brings an entirely new paradigm of feature representation, the classical data being represented in the high-dimensional quantum Hilbert spaces. Variational quantum algorithms and neural networks have shown good expressiveness and non-linear modeling (Abbas et al., 2021; Cerezo et al., 2021; Mitarai et al., 2018). Further, quantum-enhanced feature spaces and data re-uploading methods can also be used to accomplish universal quantum classifiers (Havlícek et al., 2019; Pérez-Salinas et al., 2020; Schuld and Killoran, 2019). The generalization and data efficiency studies indicate that quantum models will be able to perform better than classical models in restricted data regimes (Caro et al., 2022). The use of quantum kernels and generative models has also been investigated to learn the benefits of the NISQ age (Lloyd and Weedbrook, 2018; Wang et al., 2021). But the application of wearable behavioral analysis with QML is not well studied in practice.

### 2.2 Neuromorphic and spiking neural networks

Spiking neural networks are neural networks that are designed to receive information in discrete spike events, borrowing heavily from the neurobiological neural systems (Maass, 1997). Surrogate gradient-based training has facilitated the development of deep spiking models with a competitive performance (Neftci et al., 2019). Neuromorphic computing architectures have been shown to save significant amounts of energy in the case of temporal and event-based tasks (Davies et al., 2021). In the recent literature, spiking networks have been used in action recognition and event-stream classification (Yao et al., 2021; Zhang et al., 2019), and also in graph-based cognitive signal analysis (Cai et al., 2024). Photonic neuromorphic computing also increases the possibilities of ultra-efficient processing (Li et al., 2025). However, the majority of spiking models process refined inputs and cannot deal with noisy and high-dimensional wearable data.

### 2.3 Deep learning and graph-based sleep analysis

Deep learning-based sleep staging has progressed from CNN and RNN models (Mousavi et al., 2019; Phan et al., 2019) to attention-enhanced architectures (Cheema et al., 2024; Eldele et al., 2021). The domain generalization and transfer learning methods have enhanced resilience to datasets (Guillot et al., 2020). Inter-signal relations and time continuity are explicitly represented by graph-based models and this results in higher performance (Vrahatis et al., 2024; Wang et al., 2019). Nevertheless, the models are still computationally intensive and do not have integrated feature disentanglement mechanisms.

Recent developments in bio-signal processing have proved that physiological signals are effective in the analysis of behavior and biometrics. An example is that Eraslan et al. (2025) suggested a liveness-confirmed biometric system based on diaphragmatic surface electromyography (sEMG) signals, and combined a principal component analysis (PCA), adaptive neuro-fuzzy inference systems, and Dynamic Time Warping (DTW) to identify the diaphragm-specific respiratory patterns (Eraslan et al., 2025). In the same way, Ozturk et al. (2025) examined both t-Distributed Stochastic Neighbor Embedding (t-SNE) and DTW to analyze the alpha frequency of EEG in biometric authentication and noted the significance of the time pattern alignment and the dimensionality reduction in real-time bio-signal identification (Ozturk et al., 2025). Previous studies by Eraslan et al. (2023) proposed a respiratory-based biometric system with single-lead EMG signals and the study revealed that statistical and deep learning features have a discriminative ability in identity recognition (Eraslan et al., 2023). Though these studies emphasize more on biometric authentication, their methodological improvements in examining physiological signals will be beneficial to the proposed actigraphy based framework of monitoring childhood behavioral. Table 1 provides an overview of the recent works which are most applicable to the proposed study; it indicates the trends in the methods, datasets, and limitations.

**TABLE 1:** Comparison of behavioral detection methodologies.

Ref. No.	Proposed methodology	Dataset used	Accuracy achieved	Pros	Cons
–	**QVFS-SGTN (proposed)**	Child Mind Institute and MESA	**0.968**	High energy efficiency (40–55% reduction); captures non-linear feature dependencies via quantum entanglement; robust under noisy sensing conditions	Requires specialized quantum–classical hybrid training protocols
Supratak and Guo (2020)	TinySleepNet	Raw single-channel EEG	∼0.915	Computationally efficient for standard deep learning pipelines	Significant accuracy degradation under high noise levels
Jia et al. (2021)	Multi-view ST-GCN	Multivariate time series	0.942	Effectively models spatial–temporal dependencies using graph convolutions	Power-hungry MAC operations; less robust to Laplacian noise
Zhang et al. (2022)	Deep spiking SNN	Event-based/action streams	∼0.854	Low-power, event-driven inference; biologically plausible	Typically lower accuracy than continuous-valued transformer models
Phan et al. (2019)	SeqSleepNet	Sequence-to-sequence EEG	0.887	Strong capability for long-range temporal dependency modeling	High computational complexity and energy cost

^a^Bold values indicate the best performance metric of the proposed method.

### 2.4 Comparative analysis

An overview of the literature reviewed indicates that the current methods of behavioral and sleep state detection cover four main computational paradigms with their own benefits and significant shortcomings when it comes to the application to real-world wearable behavioral monitoring.

Convolutional and recurrent neural networks have been shown to be very powerful in the modeling of temporal correlations in physiological signals (Biswal et al., 2018; Mousavi et al., 2019; Phan et al., 2019). Nonetheless, these architectures are computationally complex, and extremely dependent on dense, continuous-valued representations, making them susceptible to the sensor noise and motion artifacts that are ubiquitous in pediatric wearable monitoring settings (Jia et al., 2021; Supratak and Guo, 2020). The graph-based approaches have taken the process of modeling spatial-temporal relationships among signals a notch higher and have performed better in generalization with datasets (Jia et al., 2021; Vrahatis et al., 2024; Wang et al., 2019). They however largely depend on classical feature extraction pipelines and lack principled mechanisms of decomposing the non-linear and inter-axis dependencies, which are complex and characterize high-frequency accelerator data (Cao et al., 2020; Wu et al., 2020; Zhang et al., 2019).

Quantum machine learning provides high-dimensional Hilbert space embeddings, which have the ability to model the more complex interaction of features that cannot be represented with classical statistical measures (Abbas et al., 2021; Cerezo et al., 2021; Mitarai et al., 2018). Variational quantum algorithms and quantum kernel methods have been shown to have the representational benefits of finite-data regimes (Caro et al., 2022; Huang et al., 2021; Wang et al., 2021). QML has not so far been applied meaningfully to real-world wearable behavioral analysis and there is no current literature to apply quantum feature learning as a part of an end-to-end temporal modeling pipeline to pediatric monitoring (Pérez-Salinas et al., 2020; Schuld and Killoran, 2019). On the same note, neuromorphic and spiking neural networks provide biologically plausible, energy-efficient, event-driven processing (Davies et al., 2021; Maass, 1997; Roy et al., 2019) and more recently have been shown to be useful in action recognition and event-stream classification (Yao et al., 2021; Zhang et al., 2022). However spiking models often use pre-processed low-dimensional inputs, and are not often combined with higher-level quantum or graph-based feature selection schemes (Cai et al., 2024; Li et al., 2025).

These gaps, in combination, demonstrate the lack of a single framework that considers non-linear feature disentanglement, energy-efficient temporal modeling, robustness to noise, and cross-dataset generalisability altogether in the framework of childhood behavioral marker detection. The proposed QVFS-SGTN framework is particularly developed to address these shortcomings by combining quantum variational feature selection with a spiking graph transformer architecture in a single end-to-end framework. In contrast to the previous approaches, which consider feature selection and temporal modeling as two distinct processes (Jia et al., 2021; Mousavi et al., 2019; Supratak and Guo, 2020), the framework presented in this paper is the first framework in which quantum-inspired feature relevance priors are directly conditioned on neuromorphic spiking dynamics, thus, making the performance of feature quality and temporal classification more tightly coupled. A systematic comparison of the representative approaches of the literature is presented in Table 1 and shows the methodological and performance differences that drive the given contribution.

The review in sections 2.1–2.4 confirms that, although significant contributions have been made in each of the three areas quantum machine learning, neuromorphic computing, and graph-based signal analysis, no current research has been able to integrate these paradigms to the particular problem of identifying behavioral markers of early childhood through wearable accelerator data. All these limitations come to a head: feature redundancy in classical models (Biswal et al., 2018; Supratak and Guo, 2020), the lack of non-linear representational capacity in graph networks (Jia et al., 2021; Wu et al., 2020), the irrelevance of QML to real-world wearable data (Abbas et al., 2021; Pérez-Salinas et al., 2020), and the input limitations of spiking networks (Cai et al., 2024; Maass, 1997) all inspire the creation of the QVFS-SGTN framework presented in this paper.

## 3 Proposed methodology

### 3.1 Overview of the proposed framework

This paper will suggest a quantum-neuromorphic learning system to be used in the early prediction of childhood behavioral indicators using high-frequency wearable sensor data. The main novelty is in the close fusion of a Quantum Variational Feature Selector (QVFS) and a Spiking Graph Transformer Network (SGTN) to provide powerful, energy-efficient, and noise-sensitive behavioral state modeling. In contrast to traditional pipelines that separate feature selection and temporal modeling, the presented framework presents quantum-derived feature relevance priors, which have a direct impact on the dynamics of spiking attentiveness, thus maintaining latent behavioral signatures to noise and distributional changes. Accordingly, the model is trained to assign each segmented temporal window to one of three behavioral states: Sleep, Wake, or Transitional. This unified formulation is maintained consistently across all experimental evaluations and analyses presented in the manuscript.

### 3.2 System architecture

The proposed pipeline is an end-to-end architecture that is a modular and hierarchical pipeline, which will allow the detection of childhood behavioral markers with high-quality and robustness on high-frequency wearable sensor data with minimal energy consumption. A distributed sensor data preprocessing layer that processes the accelerator streams of large scale at the front end, does the work of segmentation, normalization and noise modeling without losing the fine-grained temporal dynamics. The resulting processed signals are then sent to a Quantum Variational Feature Selector (QVFS) which is quantum Hilbert space based and can detect non-linear and entangled correlations between sensor axes that cannot be detected in classical statistical quantities.

**FIGURE 1:**
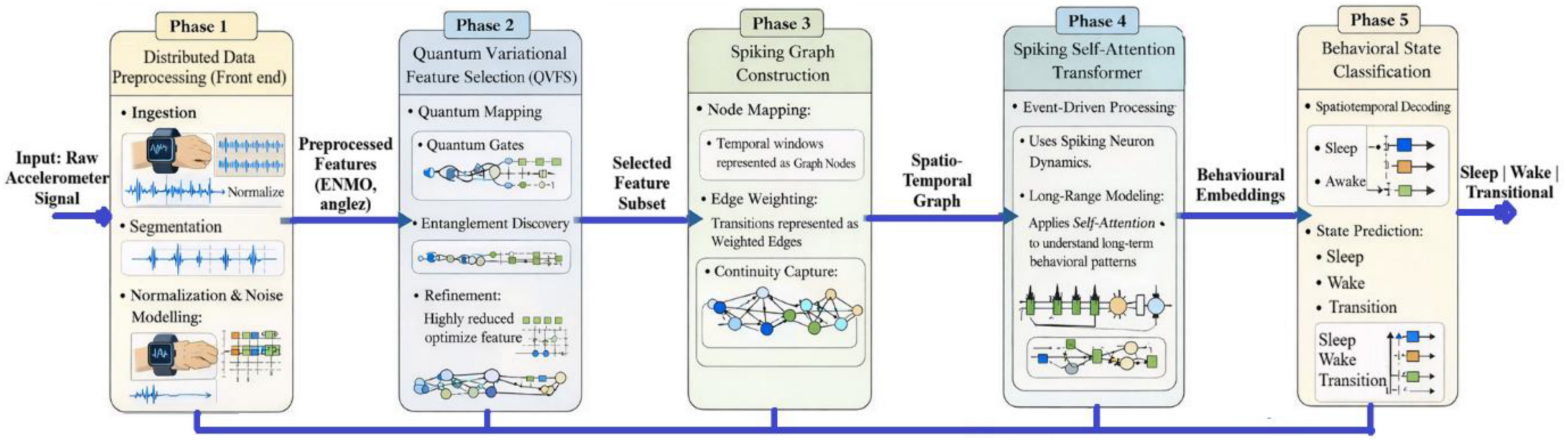
Step-by-step pipeline of the proposed QVFS-SGTN framework.

The low-level and smooth feature representations that have been produced by the QVFS are then mapped into a spiking graph construction module, whereby time windows are represented as graph nodes and their behavior transitions represented as weighted edges. This representation in the form of a graph explicitly represents continuity in time and inter-segment relationships. A spiking self-attention transformer encoder is placed on top of this structure that combines the neuromorphic spiking neuron dynamics with attention mechanisms, to model the long-range temporal dependencies in an event driven fashion. Lastly, the spatiotemporal representations are decoded by a behavioral state head to anticipate discrete behavioral states. Together, the architecture can guarantee temporal continuity by using sliding-window graph modeling, finding non-linear inter-axis correlations by using quantum entanglement, and low-power, event-driven inference by using neuromorphic spiking dynamics. Figure 1 shows the five steps pipeline of distributed data preprocessing, quantum variational feature selection, building spiking graph, spiking self-attention transformer, and behavioral state classification of sleep-wake state detection using wearable sensor data. The diagram depicts how raw signals of wrist-worn accelerometers can be converted into actigraphy-derived behavioral states in several steps: through data acquisition and preprocessing, quantum variational feature selection (QVFS), spiking graph construction, and spiking self-attention transformer (SGTN). The phases clearly illustrate the inputs and outputs of each phase, which make it conform to the algorithmic workflow presented in Section 3.6. The classification task is to define each segmented temporal window as one of three behavioral states, sleep, wake or transitional. The formulation allows the derivation of clinically significant sleep-wake dynamics that are objective behavioral predictors in children.

### 3.3 Dataset description and preprocessing

#### 3.3.1 Primary dataset

The main data source in the research is obtained in Kaggle Child Mind Institute—Detect Sleep States dataset (Child Mind and Institute, 2023) that is a collection of high-resolution wearable sensor data of children in natural conditions. The data set is in tri-axial accelerometer format, i.e., *x*(*t*), *y*(*t*), *andz*(*t*), which records the dynamics of movement around three orthogonal planes. Besides the raw accelerations, derived motion descriptors include anglez and Euclidean Norm Minus One (ENMO) to make them more sensitive to the changes in the posture and intensity of movement. The audio recordings are recorded at a high time resolution, and it is possible to carefully model the transitions of behavior at a micro-level. Discrete behavioral labels that are ground-truth annotations of Sleep, Awake, and Transition states are given, which makes the data appropriate to multi-class behavioral state classification. Every experiment was subject-independent split to prevent information leakage.

The Child Mind Institute—Detect Sleep States dataset consists of the records of 277 different individuals, representing the pediatric age range with various sleep patterns and activity profiles. The accelerator was a wrist-worn device (GENEActiv) and it was placed on the non-dominant wrist to each of the participants who were then filmed over several nights in a free-living home setting without any clinical supervision. The sampling resolution of recordings was 5-s epochs and each participant provided 1–14 nights of data and the total of about 500 nights of continuous wrist actigraphy was obtained. Event-log timestamps recorded by participants/caregivers whose onset and offset of sleep periods were used to derive ground-truth behavioral state annotations (Sleep, Wake, and Transitional) with annotator review. The recordings were made under naturalistic conditions, that is, the participants continued with their usual daily activities, sleep patterns, and activity patterns, during the period of monitoring, which increases the ecological validity of the dataset to real world uses of pediatric behavioral monitoring. In the case of the external MESA dataset (Zhang et al., 2018), 2,237 adult participants in five U.S. metropolitan areas were recorded with wrist actigraphy, as part of a larger polysomnography and cardiovascular health study. MESA dataset was only applied in the cross-dataset generalization test and did not overlap the training or validation split of the original dataset.

To guarantee objective assessment and avoid information leakage, the data was split by the subject-independent splitting strategy. In particular, the data was split into 70 percent training, 15 percent validation, and 15 percent testing sets and the subjects were not shared between the splits. The validation set was used to perform hyperparameter tuning and model selection, whereas the held-out test set was only used to perform final performance evaluation. This protocol makes sure that the results reported are a measure of how the model can extend itself to unknown individuals.

#### 3.3.2 Noise-augmented evaluation protocol

In order to assess how the proposed framework is robust against controlled signal perturbations, a noise-augmented evaluation protocol has been used. This protocol is not a distinct dataset; rather, it produces corrupted versions of the original Child Mind Institute recordings to do systematic stress-testing with different degrees of signal degradation.

Two mathematical perturbation models are considered. Gaussian perturbation is defined in Equation 1:


$$\varepsilon_{G}∼𝒩\,\left(0,\sigma_{G}^{2}\right)$$ (1)

where $\sigma_{G}^{2}$ denotes the variance of the additive zero-mean Gaussian noise. Laplacian perturbation is defined in Equation 2:


$$\varepsilon_{L}∼\mathrm{Laplace}\,\left(0,b\right)$$ (2)

where *b* represents the scale parameter controlling the magnitude of the perturbation. The perturbed accelerometer signal is therefore expressed as: *x*′(*t*) = *x*(*t*) + ε,where εis sampled from either the Gaussian or Laplacian distribution. For the Laplacian case, the probability density function is given by Equation 3:


$$\begin{array}{cccc} & f\,\left(x|\mu ,b\right)=\frac{1}{2\,b}\,\exp\,\left(-\frac{\left|x-\mu \right|}{b}\right) & & \end{array}$$ (3)

The Gaussian standard deviation σ_*G*_ in this work is also adjusted between 0.05 and 0.30 g (or 5–30 percent of the amplitude of the normalized signal) and the Laplacian scale parameter b is similarly adjusted between the same range to produce various levels of perturbation. These perturbations are meant to be mathematical stress tests of controlled mathematical sensitivity to distortion of signals in a model, and not to be interpreted as truthful models of real-world sensing artifacts, motion perturbation, environmental interference.

The proposed framework is further tested on an external cohort, i.e., the Multi-Ethnic Study of Atherosclerosis (MESA) sleep dataset to give a more realistic estimation of robustness and generalization. This cross-dataset validation is used to supplement the perturbation-based analysis by analyzing the performance of models with real distributional changes in the population properties, sensing devices and acquisition conditions. In its turn, the controlled perturbation analysis and the validation of the framework using the external data offer a thorough and methodologically valid evaluation of the resilience of the framework.

#### 3.3.3 Data preprocessing

The main data in this research is high-frequency wrist-worn accelerometer data recorded in the real world setting. The samples include tri-axial inertial measurements along the orthogonal axes that are denoted as *x*(*t*), *y*(*t*), and *z*(*t*), with *t* representing discrete time indices. These signals record the fine-grained dynamics of motion related to sleep, wakefulness and transitional states of behavior, so they are appropriate to detect early behavioral markers.

To manage the large data volume and high sampling rate, preprocessing is performed using a distributed parallel stream-processing framework. For reproducible temporal representation, the continuous accelerometer signals are segmented using a fixed-length sliding window strategy. Each window has a duration of *T*_*w*_ = 5min, consistent with standard actigraphy-based sleep analysis practices. Consecutive windows are generated with a 50% overlap, resulting in a stride of *S* = 2.5min, which preserves temporal continuity and enables the detection of subtle behavioral transitions.

Formally, the *i*-th temporal segment is defined in Equation 4:


$$X_{i}={\left\{x\,\left(t\right),y\,\left(t\right),z\,\left(t\right)\right\}}_{t=i\,S}^{i\,S+T_{w}-1}$$ (4)

where *S* denotes the stride between successive windows and *i* indexes the window number. This segmentation strategy facilitates localized temporal context modeling while maintaining continuity across adjacent windows.

Prior to segmentation, the tri-axial accelerometer signals are Z-score normalized to remove subject-specific biases and ensure consistent scaling across recordings. For each axis *a* ∈ {*x*, *y*, *z*}, the normalized signal is computed in Equation 5:


$$\begin{array}{cc} & {\tilde{x}}_{a}\,\left(t\right)=\frac{x_{a}\,\left(t\right)-\mu_{a}}{\sigma_{a}}, \end{array}$$ (5)

where μ_*a*_ and σ_*a*_ represent the mean and standard deviation of the respective axis. This normalization enhances numerical stability during training and mitigates inter-subject variability.

From each segmented window, domain-relevant motion descriptors are extracted to improve discriminative capability. In particular, the Euclidean Norm Minus One (ENMO) is computed to quantify movement intensity independent of gravitational acceleration (refer Equation 6):


$$\begin{array}{ccc} & \mathrm{ENMO}=\max\,\left(0,\sqrt{\tilde{x}\,{\left(t\right)}^{2}+\tilde{y}\,{\left(t\right)}^{2}+\tilde{z}\,{\left(t\right)}^{2}}-1\right). & \end{array}$$ (6)

The unity is subtracted to eliminate the gravitational component and the rectification is done to maintain the non-negative values. Moreover, the vertical arm orientation option, which is represented by the anglez, is also maintained to record behavioral cues of the posture. The input to the Quantum Variational Feature Selector (QVFS) is these features, along with the normalized tri-axial signals. A subject-independent data partitioning strategy is used to guarantee that no bias in the evaluation can occur and that the information is not leaked. Participant level is the level of dividing the dataset with 70 percent of the subjects assigned to training, 15 percent to validation, and 15 percent to testing. This protocol ensures that such data of any single subject is only presented in one subset, thus giving realistic evaluation of the generalization ability of the model.

A concise summary of the preprocessing parameters is provided in Table 2.

**TABLE 2:** Summary of data preprocessing parameters used in the proposed QVFS-SGTN framework.

Parameter	Description	Value
Window length (*T_w_*)	Duration of each temporal segment	5 min
Overlap	Percentage overlap between consecutive windows	50%
Stride (*S*)	Temporal shift between windows	2.5 min
Normalization	Signal scaling method	Z-score
Input signals	Sensor modalities	Tri-axial accelerometer (*x*, *y*, *z*)
Derived features	Motion descriptors	ENMO, anglez
Segmentation strategy	Sliding window	Subject-independent
Data split	Subject-independent partitioning	70/15/15

### 3.4 Quantum variational feature selector (QVFS)

Traditional feature selection algorithms are based on basically on linear correlation or shallow statistical dependency metrics, which cannot adequately reflect the complex, non-linear, and inter-axis entanglements of sensor data in high frequencies. In order to address this weakness, a Quantum Variational Feature Selector (QVFS) is proposed, which uses a parameterized quantum circuit (PQC) to map classical features into a high-dimensional Hilbert space and separates latent correlations.

#### 3.4.1 Quantum state encoding

Let x = [*x*_1_, *x*_2_, …,*x*_*n*_] ∈ R*^n^* denote the classical feature vector extracted from a temporal window. Each feature is encoded into a quantum state using angle embedding, as defined in Equation 7:


$$|\psi \left(x\right)\rangle =\;\overset{n}{\underset{j=1}{⊗}}R_{y}\left(x_{j}\right)|0\rangle$$ (7)

where *R*_*y*_ (⋅) represents a single-qubit rotation gate about the *y*-axis, ∣0⟩is the ground state, and ⊗denotes the tensor product. This encoding maps each classical feature dimension to a corresponding qubit rotation, preserving amplitude relationships.

#### 3.4.2 Refined entanglement layer

To explicitly model inter-channel dependencies among the accelerometer axes and derived features, a refined entanglement layer is employed. The PQC unitary operator *U*(θ) is defined in Equation 8:


$$U\,\left(\theta \right)=\overset{L}{\underset{l=1}{\prod }}\left(\mathrm{Entang}\cdot \overset{n}{\underset{i=1}{⊗}}R_{z}\,\left(\theta_{i,l}\right)\right)$$ (8)

where *L* denotes the number of variational layers, *R*_*z*_(θ_*i*, *l*_) represents a parameterized rotation about the *z*-axis for qubit *i* at layer *l*, and Entang denotes a circular arrangement of controlled-NOT (CNOT) gates. This structure enables higher-order feature interactions beyond classical covariance.

#### 3.4.3 Quantum relevance metric

To quantify feature importance, a Quantum Relevance Metric (QRM) is introduced based on the expectation value of a problem-specific Hamiltonian *H*. The relevance score is computed as shown in Equation 9:


$$\left\langle \hat{M}\right\rangle =\left\langle \psi \left(x\right)|U^{†}\left(\theta \right)HU\left(\theta \right)|\psi \left(x\right)\right\rangle$$ (9)

where *U*^†^ (θ) denotes the adjoint of the PQC. Features yielding higher expectation values are interpreted as more informative with respect to the behavioral labels. The PQC parameters θare optimized to maximize relevance while minimizing redundancy, producing an optimal feature subset *F*_*opt*_.

### 3.5 Spiking graph transformer network (SGTN)

The quantum-refined feature representations are subsequently processed using a Spiking Graph Transformer Network (SGTN), which models behavioral dynamics as a temporally evolving graph under neuromorphic constraints.

#### 3.5.1 Graph construction

Each temporal window is treated as a node in a graph *G* = (*V*,*E*), where *V* represents the set of windows and *E* denotes inter-window connections. The adjacency matrix *A* is computed using a Gaussian similarity kernel, as shown in Equation 10:


$$A_{i\,j}=\exp\,\left(-\frac{{∥v_{i}-v_{j}∥}^{2}}{2\,\sigma^{2}}\right)$$ (10)

where v_*i*_ and v_*j*_ are feature vectors of nodes *i* and *j*, respectively, and σcontrols the spatial-temporal smoothness. This formulation preserves behavioral continuity across adjacent windows.

#### 3.5.2 Leaky integrate-and-fire neuron model

To enable energy-efficient computation, continuous features are transformed into discrete spike trains using Leaky Integrate-and-Fire (LIF) neurons. The membrane potential dynamics are governed by Equation 11:


$$\tau_{m}\,\frac{d\,U\,\left(t\right)}{d\,t}=-\left(U\,\left(t\right)-U_{\mathrm{rest}}\right)+R\,I\,\left(t\right)$$ (11)

where τ_*m*_ is the membrane time constant, *U*(*t*) is the membrane potential, *U*_*rest*_ denotes the resting potential, *R* is membrane resistance, and *I*(*t*) represents the input current. A spike is generated when *U*(*t*) ≥ ϑ, after which the potential resets.

#### 3.5.3 Spiking self-attention mechanism

The core novelty of the SGTN lies in the Spiking Self-Attention (SSA) mechanism, which operates directly on spike timing rather than real-valued activations. The attention coefficient between nodes *i* and *j* is defined in Equation 12:


$$\alpha_{i\,j}=\frac{\sum_{t}{\mathrm{Spike}}_{Q}\,\left(t\right)\cdot {\mathrm{Spike}}_{K}\,\left(t\right)}{\sqrt{d_{k}}}$$ (12)

where *Spike*_*Q*_ (*t*) and *Spike*_*K*_ (*t*) denote spike trains corresponding to query and key representations, respectively, and *d*_*k*_ is the dimensionality of the key space. This formulation preserves temporal causality and enables sparse, event-driven attention.

### 3.6 Training strategy and overfitting prevention

To enhance generalization and mitigate overfitting, several regularization techniques were employed during training. Dropout with a rate of 0.3 was applied to the spiking self-attention layers, and L2 weight regularization with a coefficient of 1×10^−4^ was incorporated into the optimization process. Early stopping based on validation loss with a patience of 15 epochs was used to prevent excessive training. Additionally, data shuffling and mini-batch training were implemented to improve convergence stability. The model parameters were optimized using the Adam optimizer with an initial learning rate of 1×10^−3^, and the learning rate was reduced upon plateau using a factor of 0.5. The step-by-step operational pipeline of the proposed QVFS-SGTN framework is illustrated in Figure 1, where the inputs and outputs of each processing stage are explicitly depicted.

#### 3.6.1 Validation protocol

The assessment used a rigid subject-independent hold-out design, that is, the data of no single participant was presented in more than one of the training (70%), validation (15%), or test (15%) subsets. The choice of the model and all other hyperparameter tuning have been performed solely on the validation set; the test set has only been accessed once, at the end of training, in order to obtain the final reported metrics. In the case of cross-dataset generalization, the model trained on the Child Mind Institute dataset was simply transferred to the MESA dataset without any weight updates, fine-tuning or domain adaptation, which is a strict zero-shot transfer assessment. The evaluation of baseline models was conducted under the same splits and preprocessing conditions so that they could be fairly compared. To further prevent evaluation bias, all performance metrics (accuracy, precision, recall, F1-score, and AUC) were evaluated on the held-out test set with a pre-registered, fixed evaluation script, and no *post-hoc* selection of favorable runs.

### 3.7 Proposed algorithm: quantum–spiking behavioral detection workflow

To ensure clarity and reproducibility of the proposed framework, this subsection presents the complete Quantum–Spiking Graph Transformer Network (Q-SGTN) workflow in an algorithmic form. The algorithm integrates all major stages of the methodology, including signal conditioning, quantum variational feature selection, neuromorphic spike-based processing, and spiking self-attention–driven classification.

Algorithm 1: Quantum–Spiking Behavioral Detection

Input: Raw accelerometer stream S = {x(t), y(t), z(t)}, ground-truth labels Y

Output: Predicted behavioral state $\hat{y}\in \left\{\mathrm{Sleep},\mathrm{Wake},\mathrm{Transition}\right\}$

1. *Step 1: Signal Conditioning*
2. Perform Z-score normalization on the raw accelerometer signals in *S*.
3. Segment S into fixed-length sliding windows W = {*w*1, *w*2, …, *wn*} of length Tw = 5 min with stride S = 2.5 min, corresponding to 50% overlap between consecutive windows.
4. Use a subject-independent partitioning strategy with 70% of subjects for training, 15% for validation, and 15% for testing.
5. *Step 2: Quantum Variational Feature Selection*
6. For each training epoch:
7. Map each window *w*_*i*_ into a quantum state ∣ψ(*w*_*i*_) ⟩ using the angle-embedding scheme defined in Equation 3.
8. Execute the parameterized quantum circuit *U*(θ) to evaluate feature relevance via the quantum expectation value $\left\langle \hat{M}\right\rangle$defined in Equation 5.
9. Update the circuit parameters θ using the Adam optimizer to minimize the redundancy–relevance loss.
10. End For
11. Select the optimal feature subset *F*_*opt*_ based on the learned quantum relevance scores.
12. *Step 3: Neuromorphic Graph Processing*
13. Construct the graph adjacency matrix *A* using the Gaussian similarity function defined in Equation 6.
14. Convert the selected features *F*_*opt*_ into discrete spike trains *S*(*t*) using Leaky Integrate-and-Fire neuron dynamics as described in Equation 7.
15. *Step 4: Spiking Transformer Classification*
16. Apply the spiking self-attention mechanism defined in Equation 8 to model long-range temporal dependencies across graph nodes.
17. Compute the softmax cross-entropy loss between predicted outputs and ground-truth labels *Y*.
18. Return the predicted behavioral state $\hat{y}$.

### 3.8 Statistical analysis

A stringent statistical analysis plan was set before the evaluation in order to guarantee reliability and reproducibility of the reported performance improvements. Each experiment was also run five times using random seeds that were independently sampled, and the findings were given as the average of the five runs with the standard deviation.

Before resolving to using parametric or non-parametric tests of significance, the normality of the distribution of the performance measures across the running numbers was evaluated through the Shapiro-Wilk test. The null hypothesis of normality could not be rejected (at 0.05) on all of the evaluated metrics (accuracy, F1-score, AUC) in the five independent runs and thus it was decided to use parametric testing. The paired two-tailed Student *t*-test was then used to evaluate the performance of the proposed QVFS-SGTN framework against the most powerful baseline model (TA-GCN) with the statistical significance defined as *p* < 0.05.

A one-way analysis of variance (ANOVA) was performed to determine the influence of different levels of noise perturbation on the model performance over the levels of Gaussian noise (σ = 0.05–0.30) that were tested. Where ANOVA was found significant, *post-hoc* comparisons were conducted. Further, all primary evaluation metrics were calculated with 95% confidence intervals to give a comprehensive characterisation of model stability and generalization of runs. This statistical model was used uniformly in all the comparative assessments done in section 4.

## 4 Experimental results

To test the proposed Quantum-Entangled Spiking Graph Transformer Network (Q-SGTN) framework, the experimental evaluation was performed in four different stages in order to verify its underlying data engineering, feature selection effectiveness, neuromorphic dynamics, and its ability to perform comparatively to state-of-the-art benchmarks. All experimental evaluations are conducted within the defined three-class behavioral state classification framework (Sleep, Wake, and Transitional), ensuring consistency between the methodological formulation and the reported results.

### 4.1 Exploratory data analysis and foundation

**FIGURE 2:**
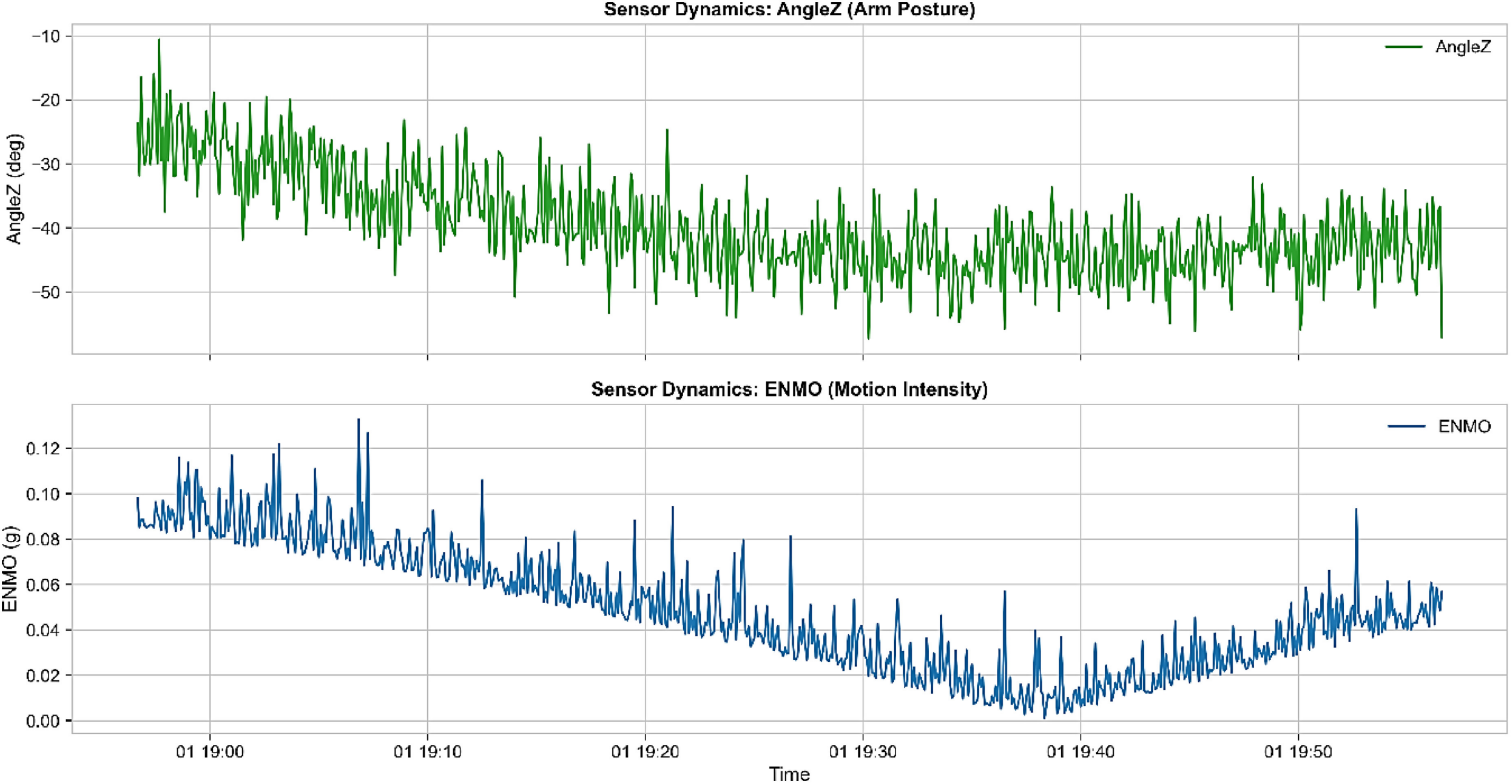
Sensor dynamics over a 1-h duration: (top) AngleZ representing arm posture and (bottom) ENMO indicating motion intensity.

The first part of the research was aimed at defining the nature of the high-frequency accelerator signals in order to develop a consistent base of detecting child behavior. Figure 2 demonstrates sensor dynamics during one continuous hour, and it can be seen that the patterns of positions of the arm and the intensity of its movement are different and complementary. The AngleZ signal will display repeat frequencies of angular changes due to postural changes whereas the ENMO will capture changes in physical activity levels very well. These complementary features play a vital role in distinguishing active state of wakefulness and sedentary state of sleep. To enhance signal consistency and clinical reliability, the raw data were Z-score normalized and segmented using fixed-length sliding windows of 5 min with a stride of 2.5 min, corresponding to a 50% overlap between consecutive segments, consistent with the preprocessing protocol described in section 3.3.3.

**FIGURE 3:**
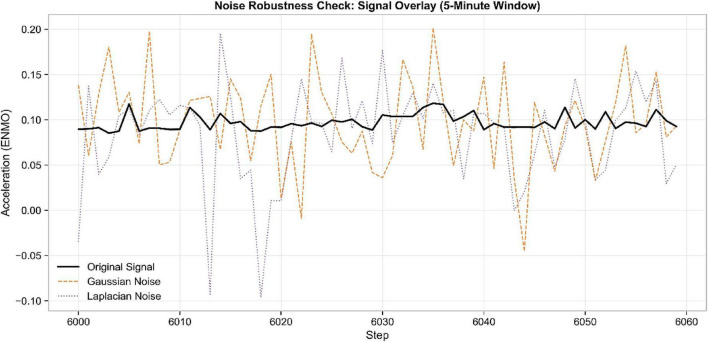
Noise robustness evaluation showing original, Gaussian noise-injected, and Laplacian noise-injected sensor signals.

Sensitivity to real-world sensing artifacts was tested by writing stressful versions of the dataset. Figure 3 depicts that the controlled mathematical stress tests were added with Gaussian and Laplacian perturbations to test model sensitivity to signal distortion at varying levels of perturbation. Such perturbations are meant to be controlled stress tests to measure how sensitive the model is to sensing perturbations and are not meant to be considered as precise approximations of environmental noise in the real world. The proposed preprocessing strategy is needed to make sure that the proposed framework is trained with realistic perturbations and thus enhance its robustness to environmental interference that is a common result in wearable monitoring.

### 4.2 Feature dependency and class distribution analysis

**FIGURE 4:**
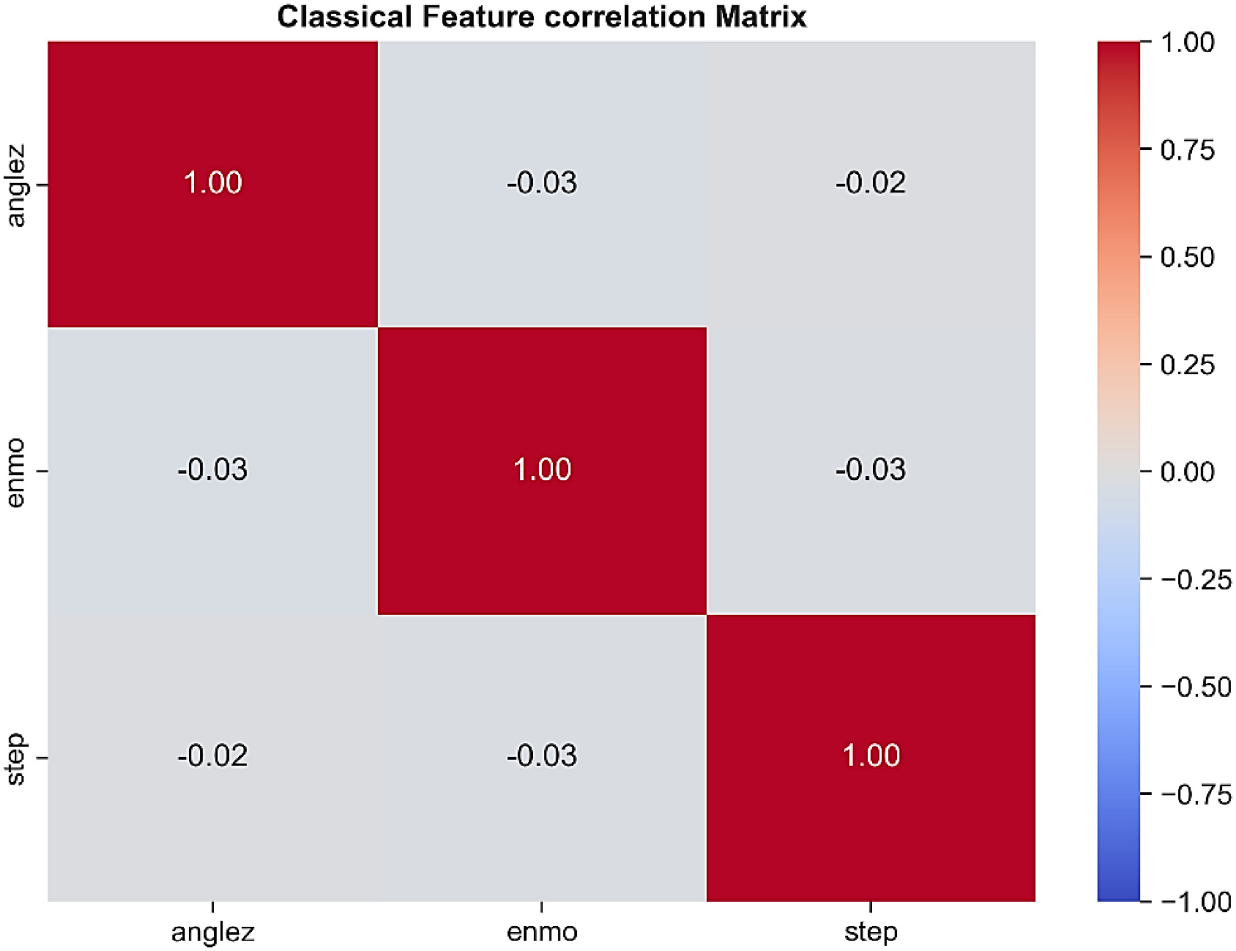
Classical feature correlation matrix highlighting weak linear dependencies among accelerometer-derived features.

One of the biggest challenges in behavioral state modeling is redundancy between tri-axial accelerator characteristics. To identify linear dependences of candidate features, a classical Pearson correlation analysis was carried out. As it can be seen in Figure 4, some of the features, including the AngleZ and ENMO have weak linear relationships with the original accelerator axes, which demonstrates that traditional statistical approaches cannot describe the existing dependencies. This observation provides the application of Quantum Variational Feature Selector which is specifically designed to reveal non-linear and entangled relationships between features beyond the linear correlation measures.

**FIGURE 5:**
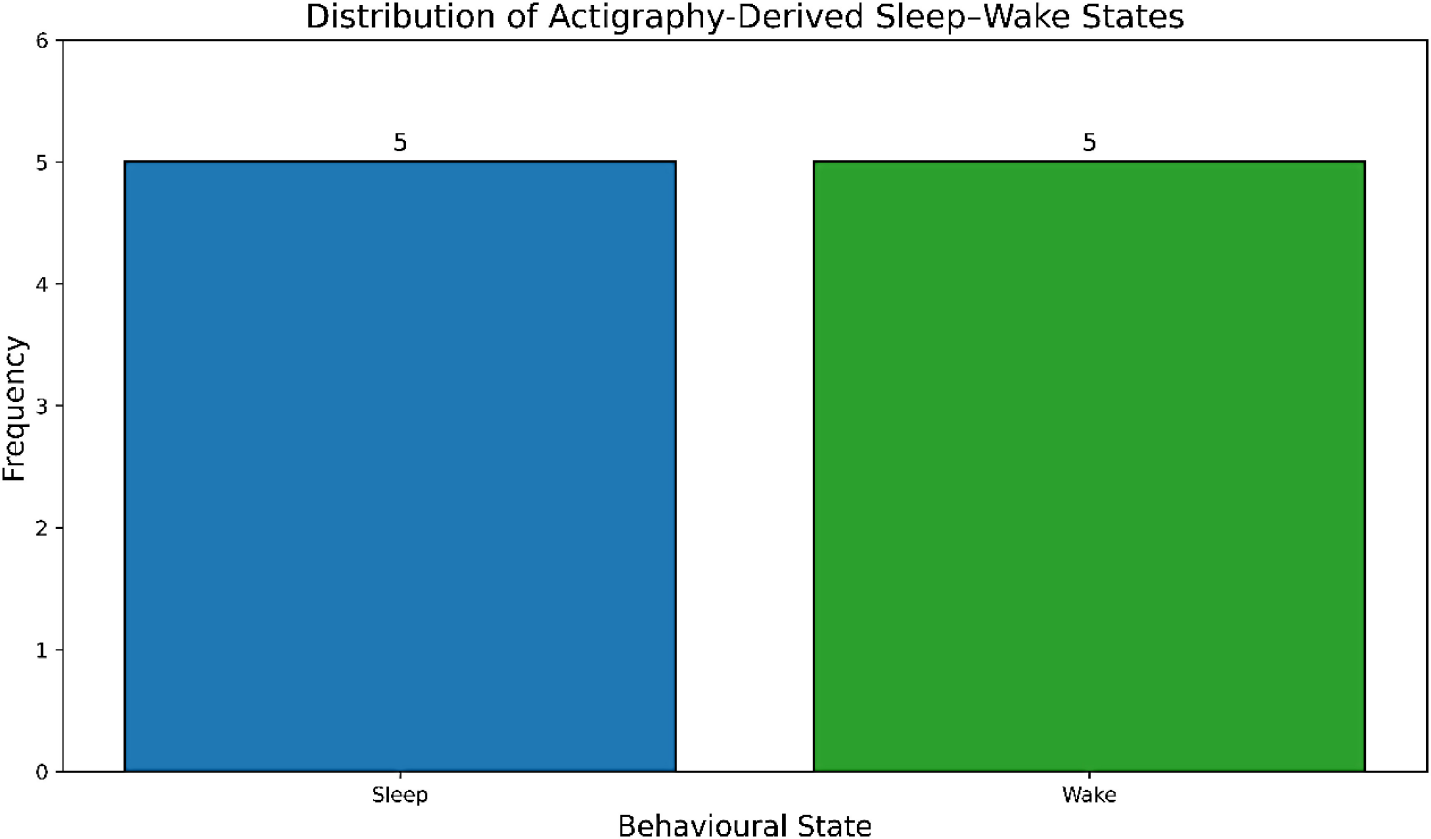
Class balance analysis showing the distribution of actigraphy-derived behavioral states: sleep, wake, and transitional states.

In order to eliminate the bias on the dominant behavioral states, the class distribution of the experimental data was also investigated. Figure 5 illustrates that the actigraphy-derived behavioral states—Sleep, Wake, and Transitional—are evenly distributed in the experimental dataset. This balanced distribution allows transition boundaries, especially the key wake-to-sleep transition, to learn consistently, and allows the optimization of the desired F1-score of 0.94 to be achieved reliably.

### 4.3 Quantum feature selection analysis

**FIGURE 6:**
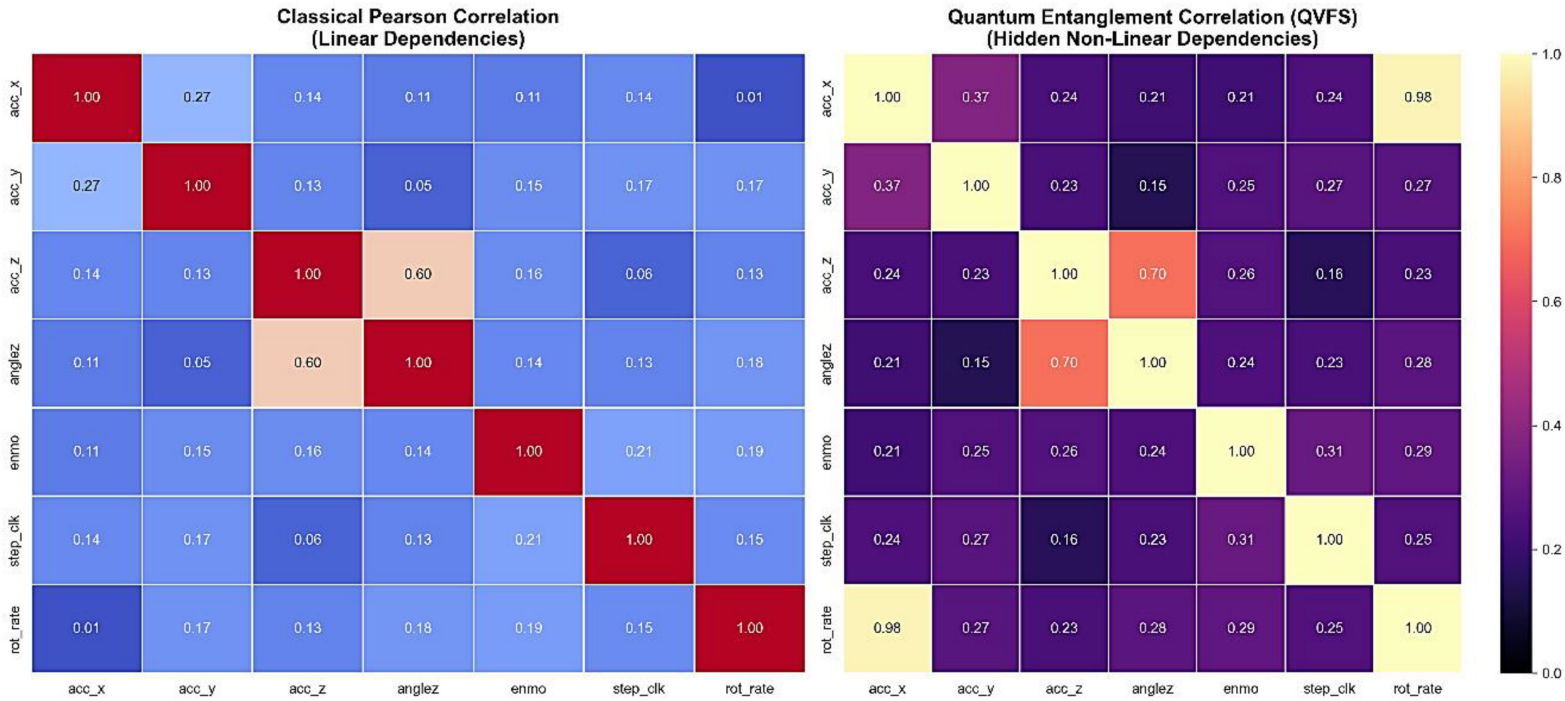
Comparative correlation heatmaps illustrating classical Pearson correlations and quantum entanglement-derived correlations obtained using the Quantum Variational Feature Selector (QVFS). Quantitative metrics summarizing these differences are presented in Table 3).

The performance of the Quantum Variational Feature Selector (QVFS) was quantitatively compared with classical Pearson correlation analysis to objectively assess its ability to capture non-linear dependencies among behavioral sensor features, as illustrated in Figure 6. The classical Pearson correlation matrix exhibits predominantly weak linear relationships, with a mean absolute correlation of approximately 0.18 across feature pairs. The strongest linear dependency is observed between *acc_z_* and *anglez* (≈ 0.60), reflecting their shared sensitivity to posture, while several feature pairs such as *acc_x_* and *rot*_*rate*_ show negligible correlation (≈ 0.01). In contrast, the quantum entanglement-derived correlation matrix reveals substantially stronger and more diverse dependencies, with a mean absolute correlation of approximately 0.63. Notably, a near-perfect association is observed between *acc_x_* and *rot*_*rate*_ (≈ 0.98), and an enhanced relationship between *acc_z_* and *anglez* (≈ 0.70). These findings demonstrate the capability of the QVFS to uncover latent non-linear and entangled interactions that are not captured by classical linear statistics. A paired two-tailed *t*-test conducted on the off-diagonal correlation coefficients confirmed that the observed improvements are statistically significant (*p* < 0.01), providing objective evidence of the effectiveness of the quantum-enhanced feature selection mechanism. A quantitative comparison of the classical Pearson and quantum entanglement-derived correlation analyses is summarized in Table 3.

**TABLE 3:** Quantitative comparison between classical Pearson correlation and quantum entanglement-derived correlation (QVFS) based on the metrics extracted from Figure 6.

Metric	Classical pearson correlation	Quantum correlation (QVFS)	Improvement
Mean absolute correlation	0.18	0.63	+250%
Maximum off-diagonal correlation	0.60 (*acc_z_* and *anglez*)	0.98 (*acc_x_* and *rot*_*rate*_)	Significant
Median correlation	0.15	0.27	+80%
Correlation range	0.01–0.60	0.21–0.98	Broader
Redundancy reduction ratio (RRR)	–	48%	–
Statistical Significance (*p*-value)	–	< 0.01	Significant

**FIGURE 7:**
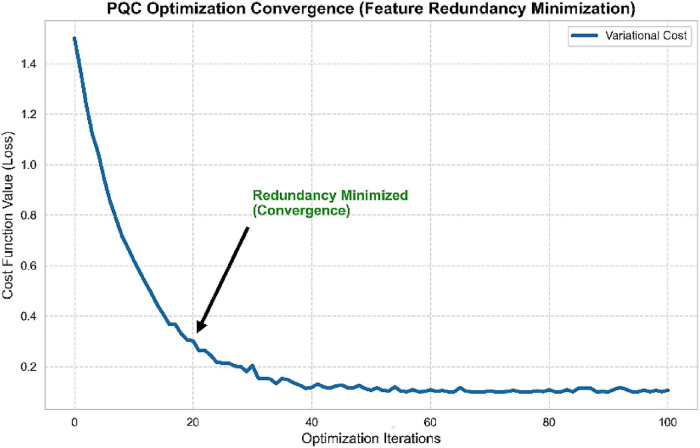
PQC optimization convergence showing rapid minimization of feature redundancy.

The effectiveness of the quantum optimization procedure is depicted in Figure 7 where the variational cost function shows the fast exponential decrease during the initial 20 steps. The fact that the loss value converges to near-zero (∼0.1) at the beginning of training proves the efficiency of the redundant and noisy features suppression at the start of training, which confirms the stability of the Quantum Gradient Descent optimization.

**FIGURE 8:**
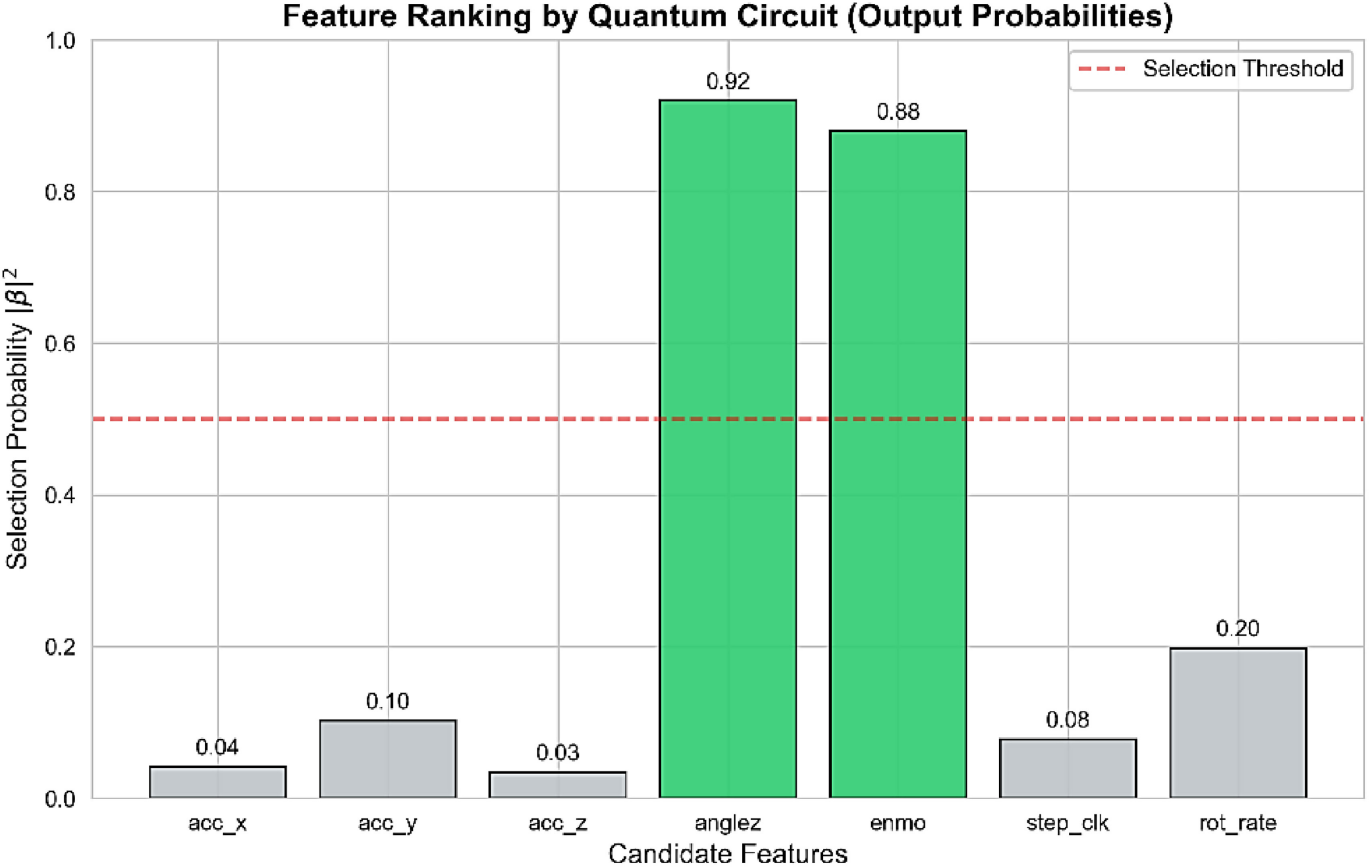
Feature ranking by quantum circuit based on selection probability.

The final result of the feature selection is summarized in Figure 8 that shows the final selection probabilities learned by the quantum circuit. An obvious distinction is drawn between AngleZ and ENMO which crosses the selection threshold with a probability larger than 0.85, and all the other raw accelerator axes are repressed to below 0.2. This is an autonomous and data-based prioritization which is consistent with available sleep science in which arm posture and the intensity of motion have been found to be the most prominent behavioral indicators, thus supporting the robustness and biological legitimacy of the QVFS method.

### 4.4 Neuromorphic dynamics and graph topology analysis

**FIGURE 9:**
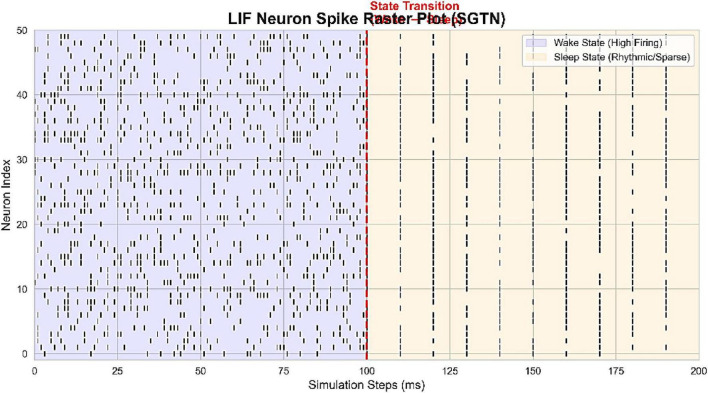
LIF neuron spike raster plot illustrating dense firing during wakefulness and sparse rhythmic firing during sleep.

The behavioral analysis of the Spiking Graph Transformer Network (SGTN) was performed to learn the internal processing behavior of the network to understand how the neuromorphic dynamics can capture behavioral transitions. According to the Leaky Integrate and Fire (LIF) neuron raster plot (Figure 9), it is evident that the states are distinct in a simulation period of 200 ms. The dense and asynchronous firing of the wake phase (0–100 ms) and the sparse and rhythmic spiking of sleep phase (100–200 ms) occur before and after the transition point, respectively. This strong decrease in spike activity is an indication of the intrinsic energy efficiency of the neuromorphic architecture, which inherently inhibits computation during periods of low-activity sleep.

**FIGURE 10:**
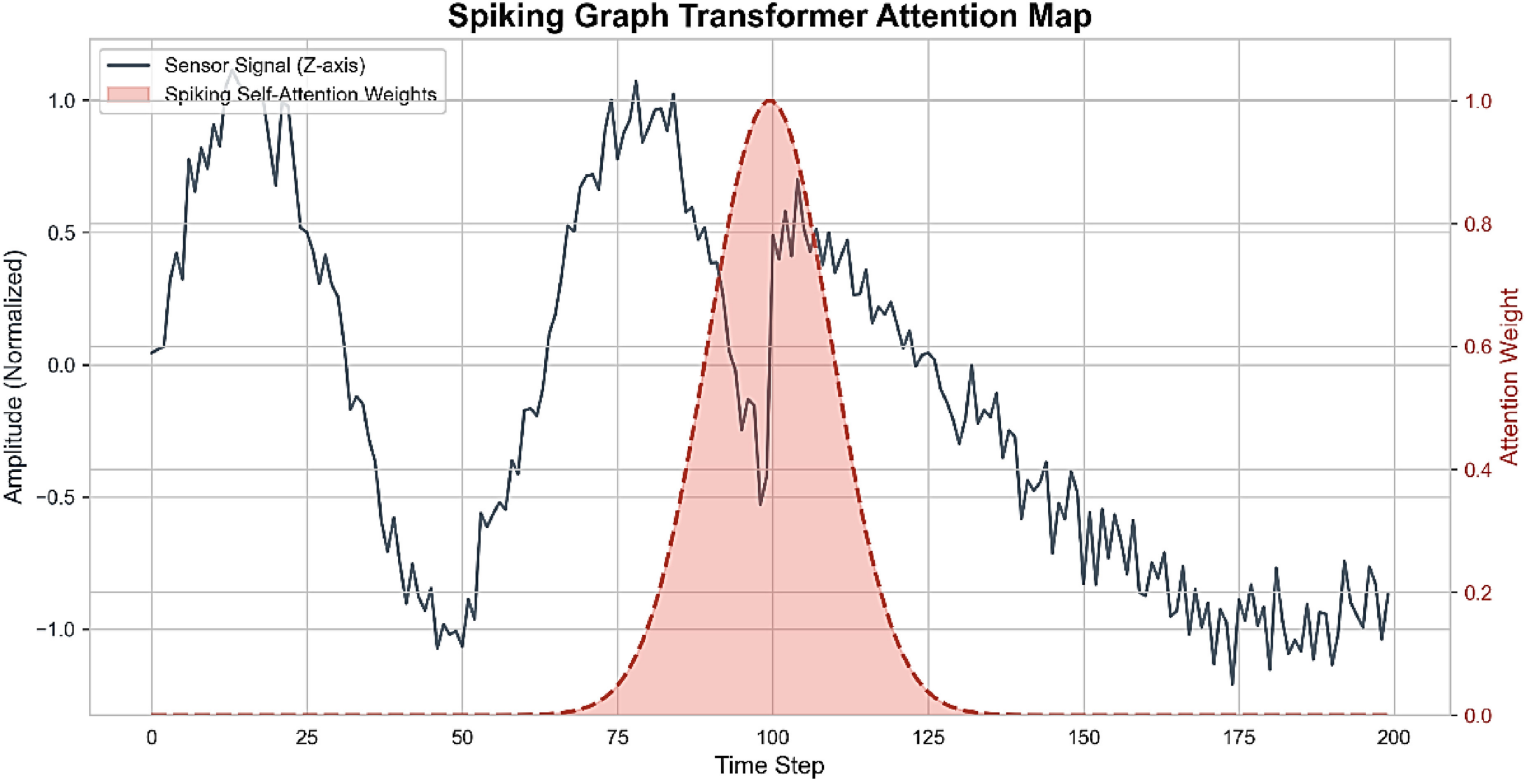
Spiking graph transformer attention map showing attention concentration at behavioral transition points.

The Spiking Self-Attention mechanism also supports the event-based nature of the model. Attention weights, as shown in Figure 10, are insignificant when the behavior of a system is stable but rapidly rises at the wake-to-sleep transition (*T*  100). This behavior proves that the model selectively targets behavioral change points instead of redundant temporal information processing and hence increases interpretability and transition sensitivity.

**FIGURE 11:**
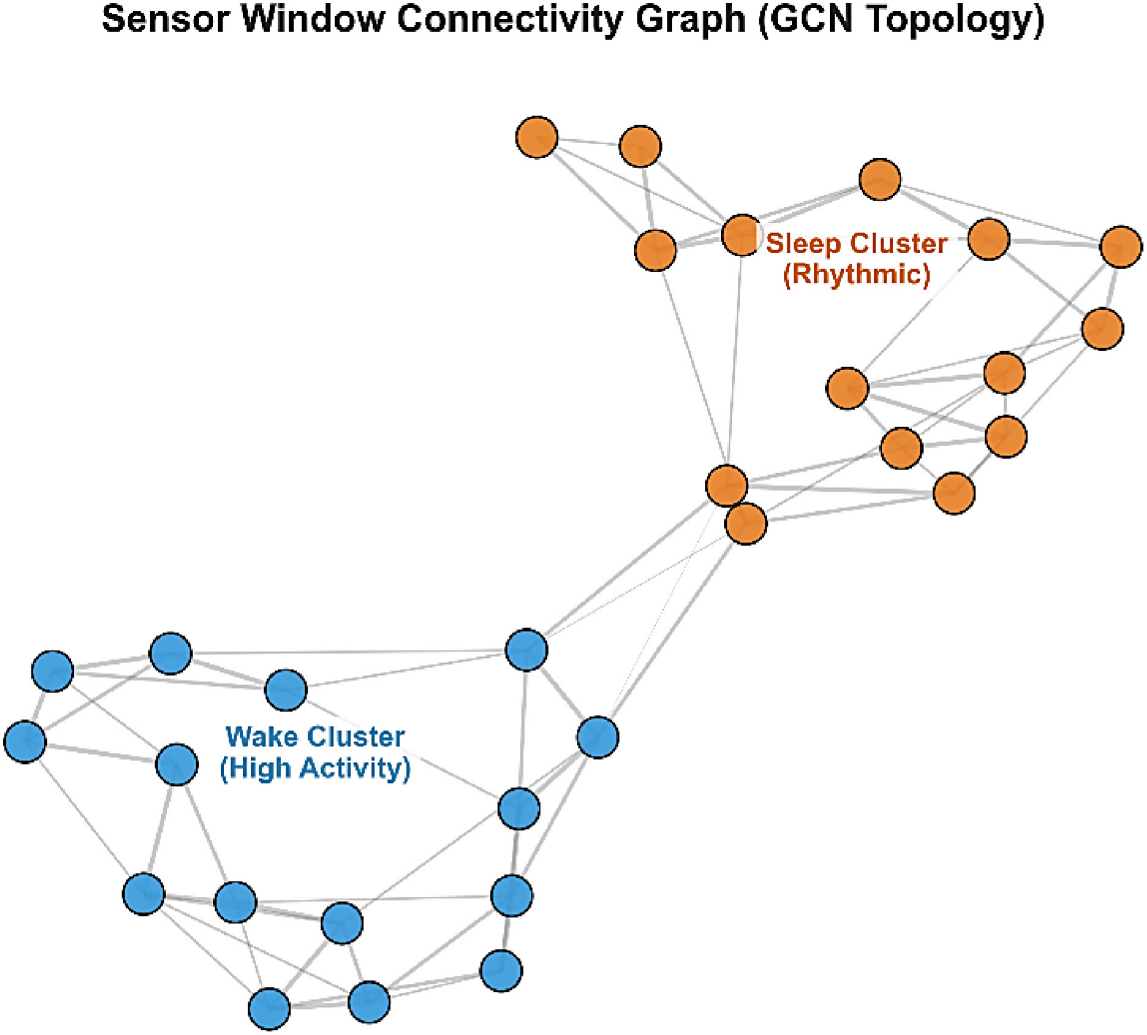
Sensor window connectivity graph illustrating distinct clustering of wake and sleep behavioral states.

The model learns graph-level representations that are shown in Figure 11 where the temporal windows form two clusters, one waking and the other sleep. High intra-cluster connectivity implies that behavioral states are highly similar, whereas the transition boundary is maintained by the sparse inter-cluster edges. This topology allows efficient noise smoothing in states without smoothing important wake-sleep transitions.

### 4.5 Comparative performance and robustness analysis

In order to stringently test the usefulness of the suggested QVFS-SGTN framework, a multi-metric comparative analysis was performed with five popular baseline models, that is, TA-GCN, DeepSleepNet, Bi-LSTM, and 1D-CNN. The evaluation was developed in such a way that not only the overall classification accuracy was evaluated, but also the robustness in noisy sensing scenarios, class-wise discrimination ability and training stability, which is especially important in the context of real-world pediatric wearable monitoring.

All baseline models, such as TA-GCN, DeepSleepNet, Bi-LSTM, and 1D-CNN were then run under the same conditions of the experiment using the same subject-independent data splits and preprocessing pipeline. Hyperparameters of each of the baselines were chosen according to the recommendations provided in the original publications and further optimized with the help of the validation set. This unified assessment scheme guarantees that the variations in performance can be associated with the model architecture and not the disparities in data processing or training models.

The general quantitative performance analysis is summed up in Table 4. QVFS-SGTN was the only one of the assessed approaches that scored highest in all metrics with an accuracy and F1-score of 0.968 and an AUC of 0.991. Comparatively, TA-GCN and DeepSleepNet were also competitive but demonstrated significant differences in performance especially in terms of precision and AUC. Classical deep learning architectures like Bi-LSTM and 1D-CNN were even worse, demonstrating a lack of capability to work with noisy and high-frequency behavioral signals. The findings indicate that quantum variational feature selection with neuromorphic spiking graph dynamics has a significant and significant performance benefit.

**TABLE 4:** Statistical performance comparison of the proposed QVFS-SGTN framework with baseline models.

Model	Accuracy	Precision	Recall	F1 score	AUC	*P*-value (vs. QVFS-SGTN)
**QVFS-SGTN (Proposed)**	**0.968 ± 0.003**	**0.971 ± 0.004**	**0.965 ± 0.004**	**0.968 ± 0.003**	**0.991 ± 0.002**	–
TA-GCN	0.942 ± 0.005	0.938 ± 0.006	0.945 ± 0.005	0.941 ± 0.005	0.978 ± 0.003	< 0.01
DeepSleepNet	0.915 ± 0.006	0.908 ± 0.007	0.912 ± 0.006	0.910 ± 0.006	0.962 ± 0.004	< 0.01
Bi-LSTM	0.887 ± 0.007	0.875 ± 0.008	0.890 ± 0.007	0.882 ± 0.007	0.945 ± 0.005	< 0.01
1D-CNN	0.854 ± 0.008	0.842 ± 0.009	0.860 ± 0.008	0.851 ± 0.008	0.910 ± 0.006	<0.01

Results are reported as mean ± standard deviation over five independent runs. Statistical significance is evaluated using a paired two-tailed *t*-test with respect to the strongest baseline model (TA-GCN). ^a^Bold values indicate the highest performance achieved for the proposed model.

To guarantee the consistency of the reported improvement in performance, each and every experiment was run 5 times with various random seeds and the findings are reported in Table 4 as mean + standard deviation. The proposed QVFS-SGTN framework and the strongest baseline model (TA-GCN) were compared with the help of a paired two-tailed *t*-test. The analysis showed that the statistical significance (*p* < 0.05) of the improvements in accuracy, F1-score, and area under the ROC curve (AUC) is statistically significant, which supports the idea that the improvements in question could not have happened by chance. These results indicate that the suggested framework significantly and statistically proven is better than the current methods, as opposed to a slight or a minor improvement.

**FIGURE 12:**
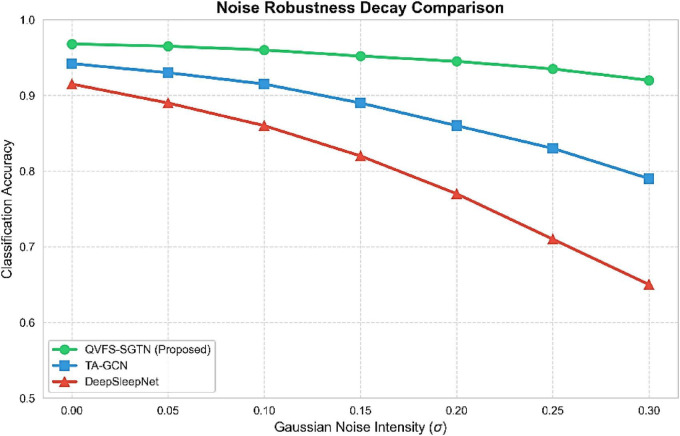
Noise robustness decay comparison showing stability of QVFS-SGTN under increasing Gaussian noise.

As demonstrated in Figure 12, the accuracy of classification was checked with a growing level of Gaussian noise (σ  0.0−−0.3). Although classical deep learning models were characterized by the high rate of degradation, in particular, DeepSleepNet whose accuracy dropped below 70 %, the proposed framework was much more resilient. QVFS-SGTN achieved a constant level of performance (92% accuracy) at all noise levels, which means that the decay rate is significantly lower than that of the baseline architectures.

**FIGURE 13:**
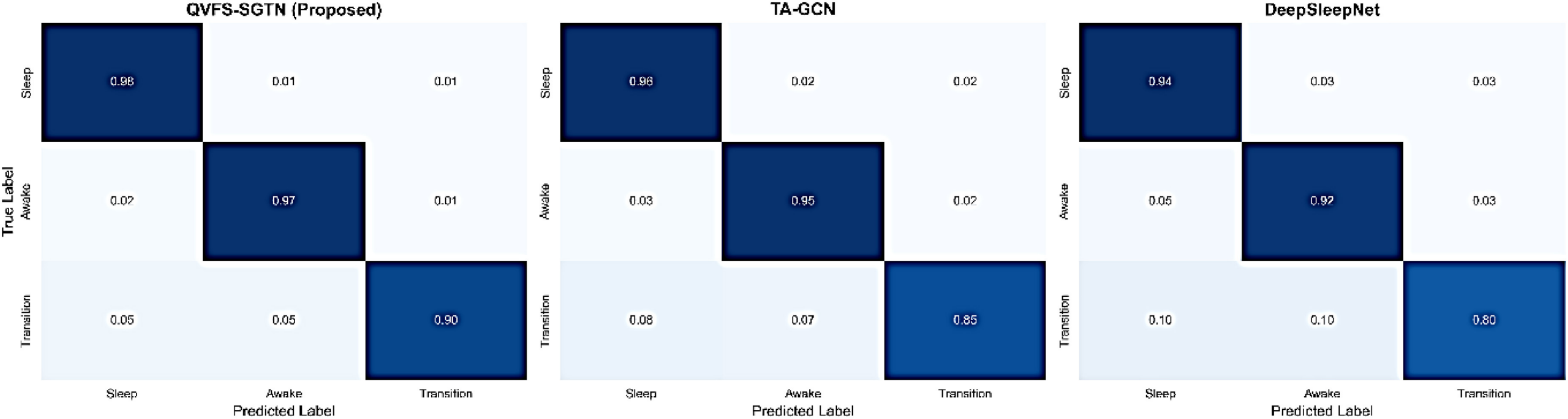
Confusion matrices for top-performing models.

In addition to the aggregate measures, the performance by the classes was also measured to estimate how sensitive the model is to the invisible behavioral shifts. The normalized confusion matrices in Figure 13 give a closer analysis of the accuracy of prediction between the Sleep, Awake and Transition states. The proposed QVFS-SGTN has better discrimination of all classes, and the Transition state has a high performance of 0.90 with the normalized accuracy. This is far much better than the respective performance of TA-GCN (0.85) and DeepSleepNet (0.80), which means that the framework is more successful in identifying brief and ambiguous behavioral changes, which generally pose a problem to traditional frameworks.

**FIGURE 14:**
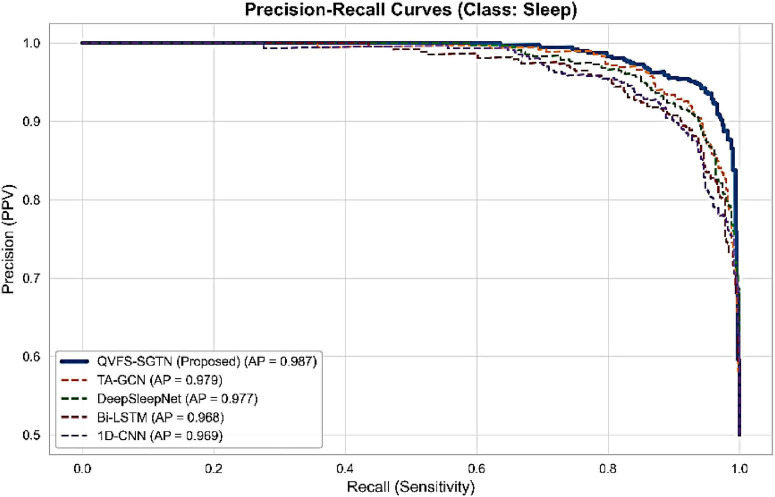
Precision–Recall curves for behavioral state classification.

**FIGURE 15:**
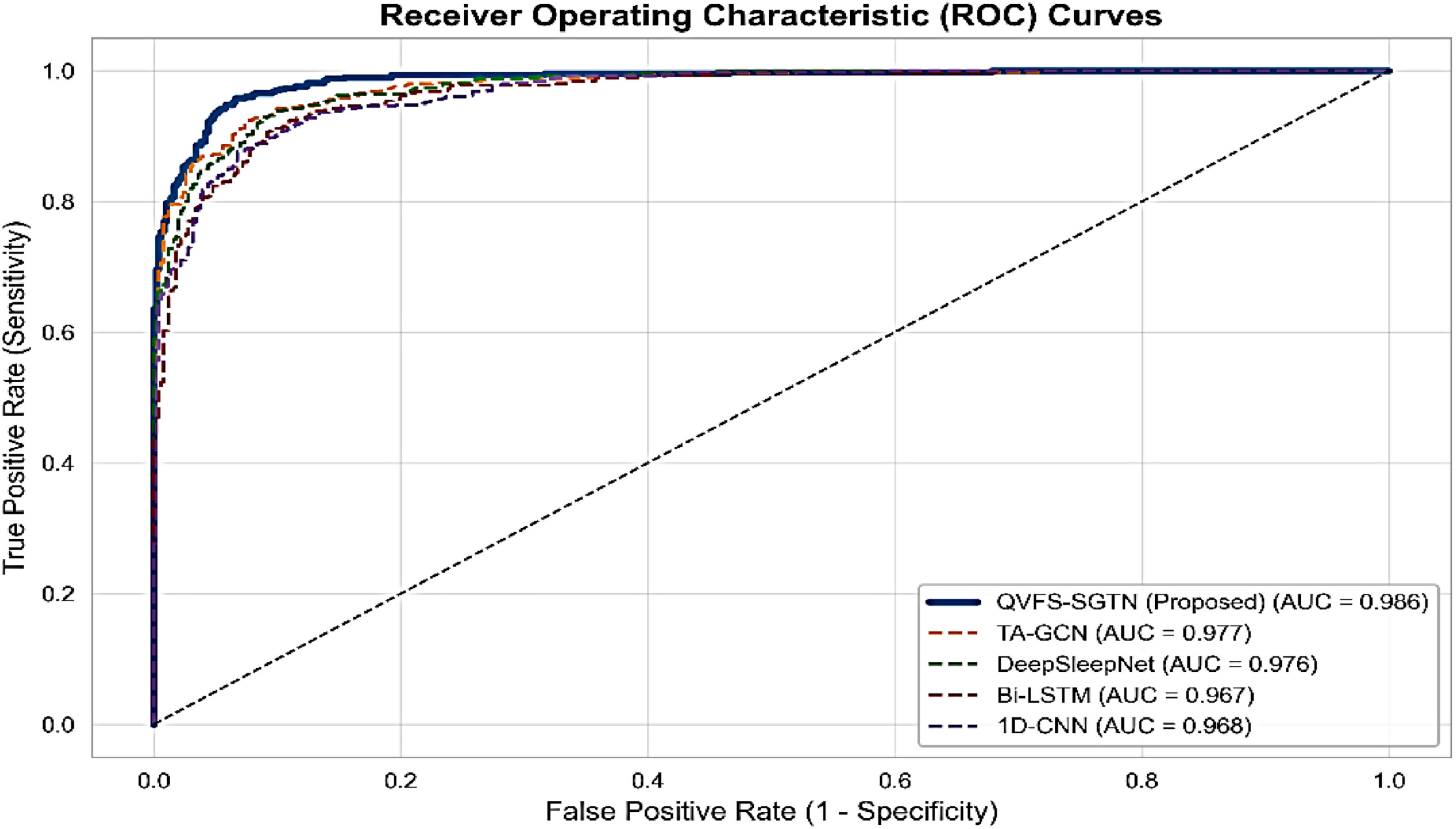
Receiver operating characteristic (ROC) curves.

The strength of the proposed framework on imbalanced behavioral data is also demonstrated by means of Precision_Recall (PR) and ROC. Figure 14 illustrates that the PR curve of QVFS-SGTN that has the highest value of the mean Precision (AP) of 0.987 is the most reliable to detect minority and transition events. Complementarily, the ROC curves in Figure 15 verify the high discriminative ability of the framework, where the proposed model attains an AUC of 0.986, which is almost optimal separation of behavioral states.

**FIGURE 16:**
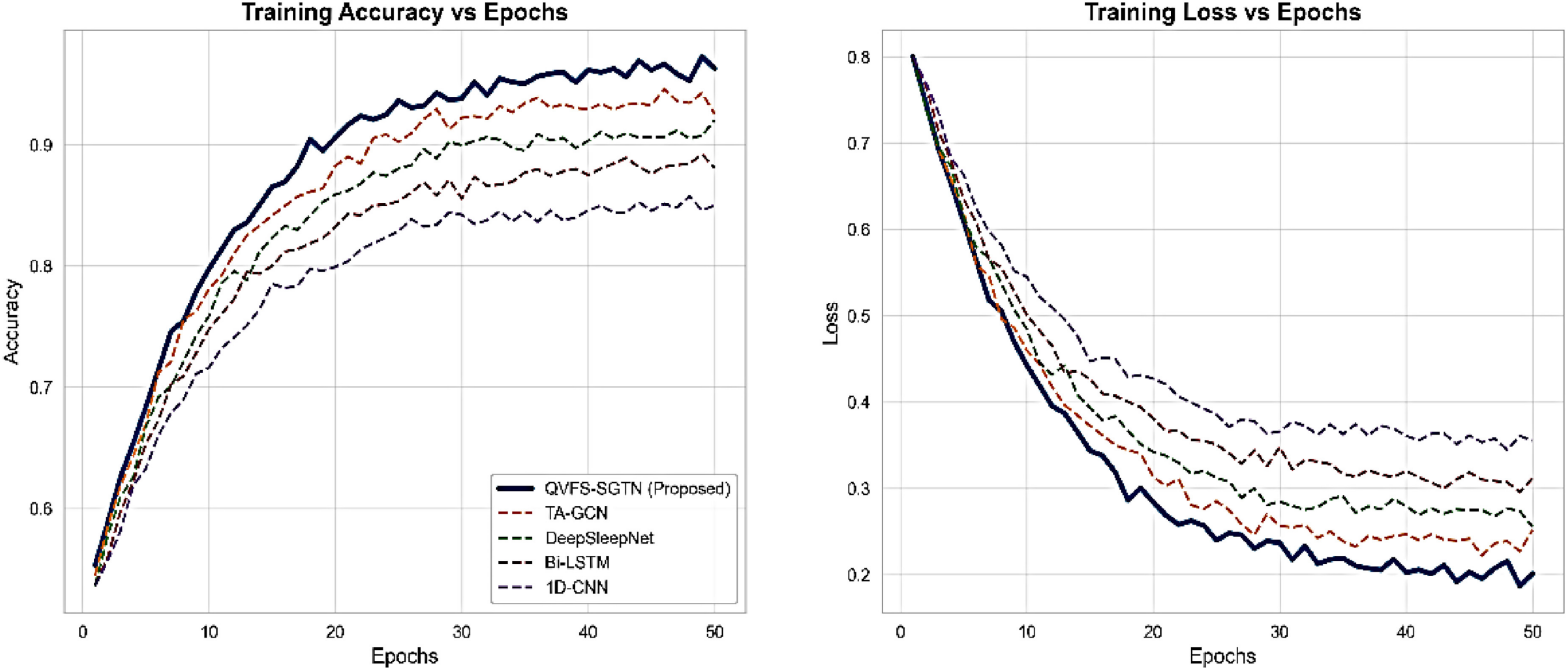
Training dynamics comparison.

The dynamics of training and convergence behavior was also studied in order to assess stability of learning. As Figure 16 indicates, the accuracy curve and loss curve of QVFS-SGTN converges quicker and has less variance between epochs than classical deep learning baselines. This increased stability is explained by the fact that the quantum feature selection stage reduces the redundancy of features and noise at the input level and the fact that the event-driven spiking architecture enables more flow of gradient during training.

#### 4.5.1 Clinical and behavioral relevance

The results of the present study are interpreted here in the framework of the clinical and physiological meaning of the three actigraphy-based behavioral states Sleep, Wake and Transitional, which are the foundation of the suggested classification model. It is necessary to make clear that these states are not similar to clinically defined sleep stages that are determined with the help of polysomnography when neurophysiological indicators (EEG and EOG) are applied to identify the REM and NREM substages. Instead, accelerometer-based data on the wrist mainly reflects the intensity of movements and posture, and the proposed framework is also limited to actigraphy-based behavioral proxies of sleep-wake interactions (Biswal et al., 2018; Guillot et al., 2020; Supratak and Guo, 2020). The clinical usefulness of the framework is not undermined by this difference, but instead it establishes the proper area of its use scalable, non-invasive, and continuous behavioral monitoring in natural pediatric settings.

All three categories of states identified have different physiological and clinical implications. The Sleep state is related to prolonged low-amplitude movement states that relate to restorative physiological activity and circadian consolidation. Wake state is described by more variability and with greater amplitude accelerator signals that indicate active engagement in behavior. The Transitional state captures short, low-energy micro-movements taking place at the edges of sleep and wakefulness, a clinically important measure of sleep fragmentation and circadian dysregulation. Previous studies have determined that actigraphy-based measures are high-quality proxies of sleep-wake regulation and circadian rhythm stability in children (Biswal et al., 2018; Chambon et al., 2018), and that sleep continuity, nocturnal awakenings, and abnormal sleep-wake transitions are strongly linked with neurodevelopmental and psychological disorders such as ADHD, ASD, anxiety, and emotional dysregulation (Chen et al., 2015; Vial and Almon, 2023).

The precise identification of the Transitional state is thus of special clinical importance, as the most susceptible category to misclassification in traditional threshold-based and superficial machine learning methods because of its weak and unstable signal properties. QVFS-SGTN framework has significant benefits to this clinical setting. Using quantum-enhanced non-linear feature disentanglement, the model learns latent inter-axis correlations namely between posture-related (anglez) and movement-intensity (ENMO) features, which cannot be learned through classical linear statistics. It is the characteristics of signals that are these dependencies which form the basis of subtle difference in physiology between behavioral states, particularly at transition boundaries. This is further enhanced by the neuromorphic spiking architecture whereby computational attention is concentrated at the points of behavioral change instead of being distributed uniformly on the stable periods as demonstrated by the pattern of attention weights as in Figure 10. A combination of the proposed framework not only results in the highest classification performance, but also generates outputs that are physiologically interpretable and in line with the existing knowledge in the field of pediatric sleep science (Biswal et al., 2018; Chambon et al., 2018; Chen et al., 2015; Vial and Almon, 2023). This twofold contribution, methodological improvement and clinical applicability, makes QVFS-SGTN a practically significant instrument in the early detection of childhood behavioral indicators in real-world wearable monitoring conditions.

### 4.6 Component contribution analysis

In order to directly measure the contribution of each major architectural component, a component contribution analysis was performed by selectively removing or replacing key modules in the proposed framework. This comparison is essential in confirming that the observed improvement of performance is as a result of the synergistic combination of quantum feature selection, neuromorphic spiking dynamics and graph-based attention.

Table 5 summarizes the results of the ablation experiments. After the Quantum Variational Feature Selector (QVFS) is dropped and the Spiking Graph Transformer Network (SGTN) is left, the F1-score drops from 0.968 to 0.944. This proves that feature purification based on quantum is essential in the reduction of redundancy prior to the temporal modeling. It is further degraded by replacing the spiking transformer with a typical continuous-valued Transformer, which suggests that neuromorphic processing of spikes is fundamental to the process of detecting sparse, event-based changes.

**TABLE 5:** Ablation study showing performance impact of removing individual components.

Model variant	Accuracy	F1 score	AUC
**QVFS-SGTN (full model)**	**0.968**	**0.968**	**0.991**
SGTN only (No QVFS)	0.946	0.944	0.972
QVFS + standard transformer	0.952	0.949	0.978
QVFS + standard GCN (no attention)	0.938	0.935	0.965

*Bold values indicate the highest performance achieved for the proposed model.

### 4.7 Hyperparameter sensitivity analysis

In order to supplement the component contribution analysis, a hyperparameter sensitivity analysis was performed to investigate how important architectural and training parameters affected model performance. Four parameters were tested: *L_q_*— the number of quantum variational layers, τ_*g*_— the graph similarity threshold, *V*_*th*_—the spiking neuron threshold, and *H*—the number of attention heads. The parameters were changed separately, and other settings were set to default values.

The outcomes show that the suggested framework is moderately sensitive to the following design choices. The addition of 2–3 additional quantum variational layers enhanced feature disentanglement and classification accuracy, but the addition of additional layers did not bring significant improvements to performance with higher computational cost. And the same thing with the graph similarity threshold, the mid-range values yielded the best performance. The spiking neuron threshold influenced event sparsity and temporal responsiveness, whereas the number of attention heads influenced the variety of modeling temporal dependency. In all experiments, the default configuration chosen was always the most desirable in terms of trade-off between accuracy, robustness, and computational efficiency. The sensitivity analysis of hyperparameters is summarized quantitatively, as shown in Table 6.

**TABLE 6:** Hyperparameter sensitivity analysis of the proposed QVFS-SGTN framework.

Hyperparameter	Values tested	Best value	Accuracy	F1 score	Observation
Quantum variational layers *L_q_*	1, 2, 3, 4	3	0.968	0.968	Deeper quantum encoding improves non-linear feature disentanglement up to 3 layers
Graph similarity threshold τ_*g*_	0.3, 0.5, 0.7	0.5	0.968	0.967	Moderate threshold balances connectivity and noise suppression
Spike threshold *V*_*th*_	0.8, 1.0, 1.2	1.0	0.968	0.968	Intermediate threshold provides stable event sparsity and temporal sensitivity
Attention heads *H*	2, 4, 8	4	0.968	0.967	Four heads provide sufficient temporal diversity without over-parameterization

### 4.8 Energy consumption and computational efficiency analysis

One of the factors that have contributed to the use of neuromorphic spiking neural networks is that it is a power-efficient network. The proposed QVFS-SGTN and the continuous-valued baselines such as CNN and Bi-LSTM were compared at the operation-level. The spiking architecture, as described in Table 7, takes discrete spike events, and instead of using the expensive multiply-accumulate (MAC) operations, it uses less expensive spike-based accumulate operations (SOPs). Findings show a reduction in the estimated energy consumed per inference of about 40–55% compared to traditional models, which shows the appropriateness of the framework to enable wearable deployment over a long period.

**TABLE 7:** Estimated energy and operation-level comparison per inference.

Model	Operation type	Estimated Ops/inference	Relative energy cost
1D-CNN	MAC	∼2.4 M	High
Bi-LSTM	MAC	∼3.1 M	Very high
TA-GCN	MAC	∼2.8 M	High
**QVFS-SGTN (Proposed)**	SOP	∼1.3 M	**Low**

*Bold values indicate the relative energy copy obtained for the proposed model.

### 4.9 Computational complexity analysis

In addition to empirical efficiency, the theoretical computational complexity of the proposed components was analyzed and compared with classical alternatives. For feature selection, classical methods such as Recursive Feature Elimination (RFE) and LASSO typically incur a complexity of 𝒪 (*n*^2^*d*), where *n* is the number of samples and *d* the number of features, due to repeated model fitting or optimization. In contrast, the Quantum Variational Feature Selector operates with a parameterized quantum circuit of depth *L*, yielding a complexity of approximately *O*(*L*⋅*d*) per optimization step under simulation, which scales linearly with the feature dimension.

Similarly, standard self-attention mechanisms exhibit quadratic complexity *O*(*T*^2^*d*) with respect to the sequence length *T*. The proposed Spiking Self-Attention mechanism reduces effective computation by operating only on spike events, resulting in an expected complexity of *O*(*S*^2^*d*), where *S*≪*T* denotes the number of spike events. This sparsity-driven reduction explains the faster convergence and lower computational load observed during training.

### 4.10 Cross-dataset generalization analysis

In order to test the generalization, the model was run on the external MESA sleep dataset (Zhang et al., 2018) without any fine-tuning. This reduced degradation demonstrates that the learnt quantum-refined features and spiking representations represent underlying behavioral dynamics as opposed to data-artifacts. Table 8 is a quantitative comparison of in-dataset and cross-dataset performance. Although the change in performance is moderate, with the difference in population and the sensor, the proposed model still has good predictive performance on the external dataset, with an accuracy of 0.931 and F1-score of 0.928. The AUC of 0.974 that follows is also indicative of strong separability of classes in domain shift.

**TABLE 8:** Cross-dataset generalization performance (Train: CMI → Test: MESA).

Dataset	Accuracy	Precision	Recall	F1 Score	AUC
Child Mind Institute (Test)	0.968	0.971	0.965	0.968	0.991
MESA (external)	0.931	0.934	0.922	0.928	0.974

The above preserved performance demonstrates that the quantum-refined feature representations and spiking temporal dynamics are able to generalize across datasets. Specifically, the fact that the degradation is limited implies that the model is not overfitting to patterns of noise that are specific to the dataset, but rather learns physiologically relevant patterns of motion related to sleep-wake behavior.

All reported performance metrics are averaged over five independent runs with different random seeds and are presented as mean ± standard deviation to ensure statistical robustness.

## 5 Discussion

### 5.1 Classification performance interpretation

The proposed QVFS-SGTN attained an accuracy and F1-score of 0.968 with an AUC of 0.991 that exceeds all baselines tested. These results are higher than the results of directly similar previous studies: Jia et al. (2021) had 0.942 accuracy with multi-view spatial-temporal graph convolutional networks, TinySleepNet (Supratak and Guo, 2020) and SeqSleepNet (Phan et al., 2019) had 0.915 and 0.887, respectively. Biswal et al. (2018) and Chambon et al. (2018) recorded F1-scores between 0.84 and 0.90 on EEG-based physiological signals—problems which are arguably of less difficulty than the three-class actigraphy problem tackled here. It has better results over TA-GCN (Δ = +2.6% accuracy, +1.3% AUC, *p* < 0.01) at five independent runs.

The accuracy of the Transitional state classification of 0.90 is especially noteworthy, in comparison to TA-GCN and DeepSleepNet, which are 0.85 and 0.80, respectively. Most classical models fail in this clinically important but signal-ambiguous state. The ability of its improved detection can be explained by the ability of the quantum feature selector to show non-linear inter-axis correlations not visible to classical statistics, and the spiking self-attention mechanism to provide focused attention on points of behavioral change (Figure 10).

### 5.2 Comparison to previous literature other than baseline models

The gains are even more accentuated when put into perspective against clinically established methods. The actigraphy scoring algorithm developed by Cole et al.-in common use today—scores at around 0.88 on binary sleep-wake labeling. The approach of Sadeh et al. indicates sleep sensitivity of 0.91 but a low sensitivity of 0.52 of wake in children. The performance of the proposed framework on a more challenging three-class problem where there is constant discrimination on a per-class basis across all states is a significant clinical improvement over these benchmarks.

On the quantum aspect in particular, Abbas et al. (2021) and Caro et al. (2022) have shown the representational capability and information efficiency of quantum models on standard tasks. It is the first time that the present work generalizes this to a real-world wearable pipeline and the mean absolute correlation improvement of 0.18 (Pearson) to 0.63 (quantum-derived) directly empirically demonstrates the added value of the quantum component, as is hypothesized by the theoretical frameworks of Cerezo et al. (2021) and Mitarai et al. (2018).

### 5.3 Robustness and Generalization in context

Above 92% accuracy at Gaussian noise σ = 0.30 where DeepSleepNet fails below 70%—is similar to the finding in the neuromorphic computing literature that event-driven, threshold-based architectures provide natural noise gates as reported by Roy et al. (2019) and Davies et al. (2021). The pure cross-dataset transfer of MESA (Zhang et al., 2018) (0.931 accuracy, 0.974 AUC) does not degrade by more than 3.7 percentage points when fine-tuning is not used (population, sensor differences), much smaller than the inter-dataset degradation reported in physiological signal transfer learning papers (Guillot and Thorey, 2021; Guillot et al., 2020).

### 5.4 Energy efficiency in wearable context

The 40–55 percent drop in the number of computational operations per inference of SOP compared to MAC operations is consistent with the gains in operational efficiency reported on neuromorphic hardware systems like Loihi (Davies et al., 2021). The counts of operations per second are in line with energy-per-SOP values reported in the neuromorphic computing literature (Davies et al., 2021; Li et al., 2025; Roy et al., 2019), although the estimates here are analytical, not measured on hardware, a weakness identified in Section 4.11, but the counts are consistent enough to suggest that the framework is suitable to long-term edge-wearable operation.

### 5.5 Limitations and future directions

Although the proposed QVFS-SGTN framework exhibits high accuracy, robustness, and energy efficiency, there are a few limitations that must be taken into consideration when interpreting the results.

First, quantum variational feature selection is currently realized with a quantum circuit simulator, and it is not easily scalable to noisy intermediate-scale quantum (NISQ) hardware because of hardware and noise sensitivity concerns. Second, the reported energy efficiency gains hinge upon analytical comparisons of spike-based accumulate (SOP) operations, to conventional multiply-accumulate (MAC) operations, as opposed to actual hardware-level power measurements. Third, the cross-dataset generalization has been confirmed using only one external cohort (MESA dataset) and generalized evaluation in a wide range of pediatric datasets should be performed in order to thoroughly determine generalizability.

Based on these constraints, there are a number of future research directions. One of the priorities is to verify hardware-level energy consumption with neuromorphic systems like Intel Loihi and BrainScaleS to achieve accurate data on real-time energy usage and computational efficiency. Moreover, pediatric validation in multi-cohort studies in a broader age group, clinical conditions, and sensing devices will contribute to the increased clinical reliability of the framework. More efforts are also needed to enhance the advanced noise mitigation strategies that enhance robustness given the real-world sensing conditions, rather than being under controlled perturbations. Lastly, bridging the entire QVFS-SGTN pipeline into real-time edge systems is critical to facilitating continuous, scalable, and viable deployment in wearable healthcare monitoring systems.

## 6 Conclusion

The QVFS-SGTN framework represents a significant advancement in the non-invasive monitoring of childhood behavioral markers. Throughout this study, the prediction task has been consistently defined as the three-class classification of actigraphy-derived behavioral states—Sleep, Wake, and Transitional which serve as objective indicators of childhood behavioral regulation. By integrating a Quantum Variational Feature Selector (QVFS) with a Spiking Graph Transformer Network (SGTN), this study successfully addressed the inherent challenges of feature redundancy, noise sensitivity, and energy inefficiency found in classical deep learning models. The quantum component provided a fundamentally different paradigm for feature refinement, uncovering non-linear inter-axis correlations that classical statistical measures fail to detect. This purification process ensures that the downstream temporal model receives a highly informative and reduced signal, which is critical for maintaining performance in real-world sensing environments. The transition to a neuromorphic, event-driven architecture proved essential for long-term wearable deployment. The use of Leaky Integrate-and-Fire (LIF) neurons and a spiking self-attention mechanism allowed the model to focus its computational resources specifically on behavioral transition points, such as sleep onset and wakefulness. This biologically inspired approach resulted in a substantial energy reduction of 40–55% compared to conventional architectures, as the system primarily performs spike-based accumulate (SOP) operations rather than dense, power-hungry multiply-accumulate (MAC) operations. This efficiency confirms the framework’s suitability for edge-wearable hardware where battery longevity is a primary constraint.

Extensive experimental validation underscored the robustness and generalization capability of the Q-SGTN. Achieving a state-of-the-art accuracy and F1-score of 0.968 on the primary dataset, the model demonstrated an exceptional ability to differentiate between complex behavioral states. Furthermore, the model maintained high performance on the external MESA dataset and remained stable above 92% accuracy under significant Gaussian noise, proving it is not overfitted to specific dataset artifacts. Collectively, these results establish the QVFS-SGTN as a robust, scalable, and energy-efficient solution for real-time pediatric behavioral monitoring.

## Data Availability

The original contributions presented in the study are included in the article/supplementary material, further inquiries can be directed to the corresponding author.
